# ﻿Taxonomic study of the genus *Erechthias* (Lepidoptera, Tineidae) from the Ogasawara Islands, with two new records and four new species

**DOI:** 10.3897/zookeys.1250.154226

**Published:** 2025-08-26

**Authors:** Jinhyeong Park, Sadahisa Yagi, Toshiya Hirowatari

**Affiliations:** 1 Entomological Laboratory, Graduate School of Bioresource and Bioenvironmental Sciences, Kyushu University, 744 Motooka, Nishi-ku, Fukuoka, 819-0395, Japan Kyushu University Fukuoka Japan; 2 Insect DX laboratory, Faculty of Agriculture, Kyushu University, 744 Motooka, Nishi-ku, Fukuoka, 819-0395, Japan Kyushu University Fukuoka Japan; 3 Insect Science and Creative Entomology Center, Kyushu University, 744 Motooka, Nishi-ku, Fukuoka, 819-0395, Japan Kyushu University Fukuoka Japan; 4 Entomological Laboratory, Faculty of Agriculture, Kyushu University, 744 Motooka, Nishi-ku, Fukuoka, 819-0395, Japan Kyushu University Fukuoka Japan

**Keywords:** Endemic species, Erechthiinae, haplotype network, Japan, oceanic island, taxonomy, Tineoidea

## Abstract

This study reviewed the genus *Erechthias* Meyrick, 1880 on the Ogasawara Islands, Japan with regards to eight recognized species, two of which were known (*E. itoi* Moriuti & Kadohara, 1994 and *E. zebrina* (Butler, 1881)), two of which are newly recorded (*E. minuscula* (Walsingham, 1897) and *E. atririvis* (Meyrick, 1931)), and four of which are new species (*E. mirabilis***sp. nov.**, *E. oculus***sp. nov.**, *E. flavimacula***sp. nov.**, and *E. nidumicola***sp. nov.**). Photographs of adult specimens and of their genitalia as well as illustrations of wing venation are provided. A preliminary phylogenetic tree based on mitochondrial DNA (the partial COI region, DNA barcode region) includes seven *Erechthias* species. Furthermore, haplotype networks were also constructed using DNA barcode regions for three species distributed across three or more island groups (Mukojima Islands, Chichijima Islands, Hahajima Islands, and other Islands). All examined *E. flavimacula***sp. nov.** and *E. nidumicola***sp. nov.** had unique haplotypes and were divided into three units corresponding to the group of islands.

## ﻿Introduction

The genus *Erechthias* Meyrick, 1880 is one of the largest groups in the family Tineidae, comprising more than 160 species (Davis and Mendel 2013). Six described species have been recorded in Japan, and two species, *E. itoi* Moriuti & Kadohara, 1994 (endemic to the Ogasawara Islands) and *E. zebrina* (Butler, 1881) (pan-global distribution), have been recorded on the Ogasawara Islands (Moriuti and Kadohara 1994).

The larvae of most *Erechthias* species are detritivorous and feed on dead plant tissues, such as dead leaves, branches, and wood bark (Robinson and Nielsen 1993). Only a few species feed on other materials such as living plant tissues, lichens, and bat guano (Robinson and Nielsen 1993; Davis and Mendel 2013; Nasu and Tamura 2020). The extreme variability in morphological characters and genital structure has complicated the taxonomy of this group (Robinson and Nielsen 1993). As a result, many monotypic genera belonging to Erechthiinae have been established, 15 of which are junior synonyms of the genus *Erechthias* (Robinson and Nielsen 1993).

The Ogasawara Islands are located approximately 1,000 km from the Japanese mainland (Tokyo) and belong to the Oceania region. Approximately 270 species of moths have been recorded on the Ogasawara Islands (Takeuchi and Ohbayashi 2006). However, the elucidation of detritivorous microlepidopteran diversity, such as that of the Tineidae, is fragmentary. In the genus *Erechthias*, an unknown species was collected from nests of a wedge-tailed shearwater, *Puffinus pacificus* (Gmelin, 1789), on Nishijima Island, Minamijima Island, and Nakodojima Island (Nasu et al. 2014). During the present study, we found eight *Erechthias* species originating from the Ogasawara Islands, including two known, two unrecorded, and four undescribed species. In this paper, we describe these based on morphological and molecular analyses and provide information on their habitat and biology.

## ﻿Materials and methods

### ﻿Sampling and dissection

Specimens were collected at night using an LED UV lamp and a mercury lamp (light trapping: LT), a Light Flight Interception trap (LTFIT), a Malaise trap (MT), and host collection. The collected host materials (fungi, wood, bird nests, and litter) were stored in food storage bags with seals or in plastic cases, and moisture was controlled by removing water drops.

The material collected in this study was deposited in the Entomological Laboratory, Kyushu University (**ELKU**). We examined specimens deposited at the following universities and museums:

**ELKU**Entomological Laboratory, Kyushu University, Fukuoka, Japan.

**NMNS**National Museum of Nature and Science, Tokyo, Japan.

**OMU**Environmental Entomology and Zoology, Osaka Metropolitan University (formerly Osaka Prefecture University), Sakai, Osaka, Japan.

Images of adults were obtained using a SONY α7R IV digital camera (SONY, Tokyo, Japan) fitted with a CANON MP-E 65 mm macro lens (CANON, Tokyo, Japan). The specimens were dissected and observed using a Nikon SMZ-U stereomicroscope (Nikon, Tokyo, Japan). To examine male and female genitalia, the abdomens of specimens were detached from the specimens and boiled in a 10% KOH solution for ~ 10 min. After washing with 70% ethanol, the genitalia were dissected in 70% ethanol and stained with a Chlorazol Black E solution. The stained abdominal pelts and genitalia were soaked in ethanol solution, and gradually increased in concentration (70–99%) to dehydrate them; they were stained at least one hour in each concentration. The dehydrated abdomen and genitalia were mounted on glass slides with Euparal. Wing venation was observed after preparation. Detached wings were cleaned to remove scales using a soft paintbrush and Kimwipes s-200 (NIPPON PAPER CRECIA CO., LTD, Tokyo, Japan) with 70–75% ethanol (EtOH) solution, stained with a mercurochrome solution for more than 1 d, dehydrated using serial dilutions of 70–99% EtOH, and mounted it in Euparal on glass slides. Genital images were captured using a Canon EOS 90D digital camera (CANON, Tokyo, Japan) connected to a Nikon ECLIPSE Ci-L stereomicroscope (Nikon, Tokyo, Japan). Photographs were processed using the Adobe Photoshop 2024 processing software.

A distribution map was created and modified using GSI maps (Geospatial Information Authority of Japan 2025) and Natural Earth (Natural Earth 2025; free vector and raster map data: https://www.naturalearthdata.com), using QGIS software v. 3.34.13 (QGIS Development Team) and Adobe Photoshop 2024 processing software. The morphological terminology generally follows that of Robinson and Nielsen (1993).

### ﻿DNA analyses

Total DNA was extracted from the abdomen and legs of adults using a DNeasy Blood and Tissue Kit (Qiagen, Venlo, the Netherlands) according to the manufacturer’s instructions. We amplified the DNA barcode region, a 658 bp fragment of the mitochondrial cytochrome *c* oxidase subunit I (COI), using the primers LepF1 (fwd) and LepR1 (rev) (Hebert et al. 2004) or LCO1490 (fwd) and HCO2198 (rev) (Folmer et al. 1994). For details of the analytical method, see Park et al. (2024a). PCR amplification by LepF1/LepR1was performed using the following thermocycling program: initial denaturation at 98 °C for 3 min, followed by 35 cycles at 98 °C for 10 s, 45 °C for 5 s, and 68 °C for 5 s. DNA barcodes were generated and deposited in the DDBJ (Suppl. material 1).

For constructing the preliminary maximum likelihood (ML) and maximum parsimony (MP) trees from the partial COI mitochondrial genes, 57 operational taxonomic units were analyzed in the general time-reversible model with Gamma distribution and invariant sites using MEGA 11.0.13 (Tamura et al. 2021). The sample data used in this study are summarized in Suppl. material 1. *Pyloetis mimosae* (Erechthiinae, used as the out group) and the unidentified Arthropoda species from British Indian Ocean Territory which matched to *Erechthias itoi*, based on the identification engine of BOLD Systems, were downloaded from the BOLD systems and also analyzed. Branch support was calculated using 1,000 bootstrap replicates. Uncorrected pairwise distances (p-distances) were also calculated using the same samples and software used for phylogenetic analyses (Suppl. material 2). To identify closely related species, the COI sequences were searched against the mitochondrial nucleotide collection of the BOLD identification system (Ratnasingham and Hebert 2007).

The haplotype networks were constructed to clarify the intraspecific genetic structures of three species (*E. itoi*, *E. nidumicola* sp. nov., *E. flavimacula* sp. nov.) with sufficient samples distributed across three or more island groups . The haplotype network analyses were conducted using the median-joining network algorithm (Bandelt et al. 1995) of the software package Network 10.2.0.0 (https://www.fluxus-engineering.com/sharenet.htm) based on the mtDNA COI barcode region. The unique haplotypes were determined using DnaSP v. 6.12.03 software (Rozas et al. 2017).

## ﻿Taxonomy

### ﻿Genus Erechthias

Taxon classificationAnimaliaLepidopteraTineidae

Meyrick, 1880

26FD4FC4-8D36-5F15-8631-E6D1FBBB3031

Erechthias Meyrick, 1880: 261. Type species: Erechthias charadrota Meyrick, 1880: 268, by subsequent designation by Meyrick 1915a: 233. Type locality: New Zealand.
Ereunetis Meyrick, 1880: 258. Type species: Ereunetis iuloptera Meyrick, 1880: 260, by subsequent designation by Walsingham 1914: 347. Type locality: Australia. Synonymized by Robinson 1983.
Decadarchis Meyrick, 1886: 290. Type species: Decadarchis melanastra Meyrick, 1886: 291, by monotypy. Type locality: Fiji. Synonymized by Robinson 1983.
Hactacma Meyrick, 1915a: 233. Type species: Erechthias chasmatias Meyrick, 1880: 263, 264, by original designation. Type locality: New Zealand. Synonymized by Dugdale 1988.
Nesoxena Meyrick, 1929: 506. Type species: Nesoxena strangulata Meyrick, 1929: 507, by monotypy. Type locality: Tuamotu Archipelago. Synonymized by Robinson 1983.
Amphisyncentris Meyrick, 1933: 412. Type species: Amphisyncentris glyphidaula Meyrick, 1933: 412, by monotypy. Type locality: Fiji. Synonymized by Robinson 1983.
Gonglyodes Turner, 1933: 180. Type species: Gonglyodes centroscia Turner, 1933: 180, by monotypy. Type locality: Australia. Synonymized by Robinson and Nielsen 1993.
Caryolestis Meyrick, 1934: 109. Type species: Caryolestis praedatrix Meyrick, 1934: 110, by monotypy. Type locality: Tahiti. Synonymized by Robinson 1983.
Triadogona Meyrick, 1937: 153. Type species: Triadogona amphileucota Meyrick, 1937: 153, by monotypy. Type locality: Fiji. Synonymized by Robinson 1983.
Anemerarcha Meyrick, 1937: 154. Type species: Anemerarcha entomaula Meyrick, 1937: 154, by monotypy. Type locality: Fiji. Synonymized by Robinson 1983.
Empaesta Bradley, 1956: 163. Type species: Tinea capnitis Turner, 1918: 288, by original designation. Type locality: Norfolk Island. Synonymized by Robinson and Nielsen 1993.
Tinexotaxa Gozmány, 1968: 306. Type species: Tinexotaxa travestita Gozmány, 1968: 306, by original designation. Type locality: Sierra Leone. Synonymized by Robinson 1983.
Acrocenotes Diakonoff, [1968]: 259, 262. Type species: Acrocenotes niphochrysa Diakonoff, [1968]: 257, 262, by original designation. Type locality: Philippines. Synonymized by Robinson and Nielsen 1993.
Neodecadarchis Zimmermann, 1978: 264, 341. Type species: Ereunetis flavistriata Walsingham, 1907: 716, by original designation. Type locality: Hawaii. Synonymized by Robinson 1983.
Lepidobregma Zimmermann, 1978: 264, 351. Type species: Ereunetis minuscula Walsingham, 1897: 155, by original designation. Type locality: West Indies. Synonymized by Robinson 1983.
Pantheus Zimmermann, 1978: 264, 353. Type species: Ereunetis pencillata Swezey, 1909: 13, by original designation. Type locality: Hawaii. Synonymized by Robinson 1983.

### ﻿Key to species of *Erechthias* occurring on Ogasawara Islands based on male genitalia

**Table:** 

1	Basicostal process present in valva	**2**
–	Basicostal process absent in valva	**3**
2	Phallus with ~15 stout cornute	***E. itoi***
–	Phallus without stout cornute	***E. oculus***
3	Dorsal costa of valva with short spines	**4**
–	Dorsal costa of valva without short spines	***E. zebrina***
4	Phallus with single robust cornutus	**5**
–	Phallus with two robust cornute	**6**
5	Apical area of saccus rounded	***E. atririvis***
–	Apical area of saccus sharp	***E. minuscula***
6	Shape of robust cornuti blade shaped	**7**
–	Shape of robust cornuti spine shaped	***E. mirabilis***
7	Apical area of valva protruded	***E. nidumicola***
–	Apical area of valva rounded	***E. flavimacula***

### ﻿Key to species of *Erechthias* occurring on Ogasawara Islands based on female genitalia

**Table:** 

1	Signum present in corpus bursae	**2**
–	Signum absent in corpus bursae	**3**
2	Ductus bursae with sclerotized area	***E. itoi***
–	Ductus bursae without sclerotized area	***E. nidumicola***
3	Segment VIII elongated	**4**
–	Segment VIII broad	**5**
4	Signum with sharp and curved lobe	***E. mirabilis***
–	Signum rounded rectangular	***E. atririvis***
5	Tergum VIII with stout dorsal rami fusing with apophysis anterioris	**6**
–	Tergum VIII with stout dorsal rami, but not fusing with apophysis anterioris	***E. oculus***
6	Posterior end of corpus bursae sclerotized	***E. flavimacula***
–	Posterior end of corpus bursae not sclerotized	**7**
7	Signum relatively large, curved	***E. zebrina***
–	Signum relatively small, not curved	***E. minuscula***

### ﻿ Erechthias itoi

Taxon classificationAnimaliaLepidopteraTineidae

Moriuti & Kadohara, 1994

45C2F15E-3819-551E-9C9B-953372A46E95

Figs 1 , 2 , 27 , 35 , 43 , 44 , 51 , 52 Japanese name: Ogasawara-tsumaorega

Erechthias itoi Moriuti & Kadohara, 1994: 567. Type locality: Japan (Chichijima Island); Yoshimatsu and Makihara 1995: 449, figs 1, 2; Sakai 2013: 130, fig. 3-12-16; Hirowatari and Yagi 2023: 24.

#### Material examined.

**Japan: [Tokyo, Ogasawara Isls.]: [Chichijima Is.**]: 1♂, Mt. Sakaigatake, alt. 390 m, 17. VI. 2023, LT, J.-H. Park leg., ELKU • 3♂, Shigureyama, alt. 252 m, 18. VI. 2022, LT, S. Tomura leg., ELKU • 1♂, same data, genitalia slide no. JP-164, DNA sample JHP-266, museum ID ELKU-I-L-Bonin 000071, ELKU • 4♂, same locality, alt. 252 m, 18. VI. 2022, S. Yagi, T. Hirowatari, S. Tomura, M. Kimura leg., ELKU • 2♂, same locality, 26. VI. 2022, LT, S. Yagi, S. Tomura, M. Kimura leg., ELKU • 10♂, same locality, 13. XI. 2022, LT, T. Hirowatari, J.-H. Park & M. Kimura leg., ELKU • 10♂1♀, same locality, 9. III. 2023, LT, T. Hirowatari, S. Yagi, Y. Matsui, S. Tomura, J.-H. Park & M. Kimura leg., ELKU • 6♂, same locality, 13. III. 2023, T. Hirowatari, S. Yagi, Y. Matsui, S. Tomura, J.-H. Park & M. Kimura leg., ELKU • 9♂, same locality, 9. III. 2023, LT, T. Hirowatari, S. Yagi, M. Kimura, S. Tomura, Y. Matsui & J.-H. Park leg., ELKU • 1♂, same data, JP-173, JHP-117, ELKU • 3♂, same data, S. Tomura leg., ELKU • 1♂1♀, same locality, 11. VI. 2023, LT, T. Hirowatari, S. Yagi, Y. Matsui, J.-H. Park, I. Kawashima, J. Hamaguchi & M. Kimura leg., ELKU • 15♂, Tatsumi road, 11. VI. 2023, LT, J.-H. Park leg., ELKU • 1♂, Higashi-machi, 12–14. III. 2023, T. Hirowatari, S. Yagi, M. Kimura, Y. Matsui & J.-H. Park leg., ELKU • 1♂, same locality, 13. III. 2023, LT, S. Yagi leg., ELKU • 1♂, same locality, 22. I. 2024, at Light, Yu Hisasue leg., ELKU • 1♂, Higashidaira, 18. VI. 2022, T. Hirowatari leg., ELKU • 1♂, same locality, 26. VI. 2022, T. Hirowatari leg., ELKU • 1♂, same locality, 15. XI. 2022, T. Hirowatari leg., ELKU • 1♀, same locality, 10. III. 2023, SW, T. Hirowatari, S. Yagi, M. Kimura, S. Tomura, Y. Matsui & J.-H. Park leg., ELKU • 1♂, Asahiyama, alt. 206 m, 18. VI. 2022, S. Tomura leg., JP-162, JHP-265, ELKU-I-L-Bonin 000070, ELKU • 1♂, same locality, 13. XI. 2022, T. Hirowatari leg., ELKU • 1♂, 11. VI. 2023, M. Kimura leg., ELKU • 4♂, same locality, 14. XI. 2022, LT, J.-H. Park, T. Hirowatari, M. Kimura leg., ELKU • 1♂, same data, JP-179, JHP-122, ELKU • 1♂, Chuosan, 14. XI. 2022. XI. 14, LT, T. Hirowatari, J.-H. Park & M. Kimura leg., ELKU • 1♂, Funamiyama, alt. 150 m, 11. III. 2022, LT, T, Hirowatari, S. Yagi, M. Kimura, S. Tomura, Y. Matsui & J.-H. Park leg., ELKU-IL Bonin 000181, ELKU • 1♂, same data, alt. 135 m, S. Tomura leg., ELKU • 1♂, Kopepe-kaigan, 12. VI. 2023, T. Hirowatari leg., ELKU • 1♂, Kitafukurozawa first tunnel, 10. III. 2023, T. Hirowatari, S. Yagi, M. Kimura, S. Tomura, Y. Matsui & J.-H. Park leg., ELKU • 1♀, Nagatani (Ogaguwanomori), 12. III. 2023, LT, T. Hirowatari, S. Yagi, Y. Matsui, S. Tomura, J.-H. Park & M. Kimura leg., JP-175, JHP-119, ELKU • 1♀, Kitafukurozawa, alt. 10 m, 26. IX. 2023, LT, Y. Matsui leg., ELKU-IL Bonin 000182, ELKU • 3♂, Mt. Akahata-yama, 13. III. 2023, LT, T. Hirowatari, S. Yagi, M. Kimura, Y. Matsui & J.-H. Park leg., ELKU • 1♂, Ogamiyama, 25. VI. 2022, LT, S. Yagi, S. Tomura leg., ELKU • 1♂, same data, S. Yagi, T. Hirowatari, S. Tomura, M. Kimura leg., ELKU • 3♂, same data, S. Tomura leg., ELKU • 1♂, same locality, 11–16. VII. 2024, MT, J.-H. Park leg., ELKU • 4♂ Mt. Mikazuki-yama, 11. III. 2023, LT, T. Hirowatari, S. Yagi, M. Kimura, Y. Matsui, S. Tomura &. J.-H. Park leg., JP-176, JHP-120, ELKU • 1♂, same locality, 13. VI. 2023, T. Hirowatari leg., ELKU • 1♀, same locality, 13. VI. 2023, J.-H. Park leg., JP-139, JHP-095, ELKU • 1♂1♀, Ohgiura-Komagari, 18–31. VII. 2023, N. Tsuji leg., ELKU • [**Hahajima Is.**]: 2♂, Motochi, 12. IV. 1999, S. Omura leg., ELKU • 1♂, same locality, 15. IV. 1999, S. Omura leg., ELKU • 1♂, same locality, Emrg. on 15. IV. 1999, S. Omura leg., ELKU • 1♂, same locality, 15. VII. 2002, S. Omura leg., ELKU • 1♂, Chokiyama, 22. VI. 2022, LT, S. Tomura leg., ELKU • 1♀, same locality, 15. VI. 2023, LT, J.-H. Park leg., JP-138, JHP-094, ELKU • 1♂, Hyogidaira, alt. 89 m, 23. VI. 2022, LT, S. Tomura leg., ELKU • 1♂, same data, JP-165, JHP-267, ELKU-I-L-Bonin 000072 • 1♂, same data, JP-167, JHP-268, ELKU-I-L-Bonin 000073, ELKU • 1♂, Mt. Funaki-yama, 17. III. 2023, LT, S. Yagi leg., ELKU • 2♂, Funakiyama, 11. XI. 2022, T. Hirowatari, J.-H. Park & M. Kimura leg., ELKU • 3♂, same locality, 17. III. 2023, LT, T. Hirowatari, S. Yagi, Y. Matsui, J.-H. Park, N. Katsube, M. Kimura leg., ELKU • 12♂3♀, Funakiyama chûfuku, 17. III. 2023, LT, J.-H. Park leg., ELKU • 1♂, Shin yûhigaoka, 7. XI. 2022, LT, J.-H. Park, T. Hirowatari, M. Kimura leg., ELKU • 2♂, same locality, 15. III. 2023, T. Hirowatari, S. Yagi, Y. Matsui, J.-H. Park, N. Katsube & M. Kimura leg., ELKU • 1♂, 14. VI. 2023, LT, J.-H. Park leg., ELKU • 1♂, Nishiura, 21. VI. 2022, LT, S. Tomura leg., ELKU • 1♀, same data, S. Yagi, T. Hirowatari, S. Tomura, M. Kimura leg., ELKU • 1♀, Minamizaki, 23. VI. 2022, SW, S. Yagi leg., ELKU • 1♂, same data, Beating: dead leaves of Livistona chinensis var. boninensis, S. Tomura leg., ELKU • 4♂, 10–11. XI. 2022, SW, J.-H. Park leg., ELKU • 1♂, same data, JP-177, JHP-121, ELKU • 1♀, Omoto bridge, 10. XI. 2022, LT, J.-H. Park, T. Hirowatari & M. Kimura leg., ELKU • 1♀, Kitakoh, 17. VI. 2023, LT, Y. Matsui leg., ELKU • [**Iwoto Is.**]: 1♂, 11. IV. 2022, L5, M. Kimura leg., JP-168, JHP-269, ELKU-I-L-Bonin 000105, ELKU.

**Figures 1–8.**
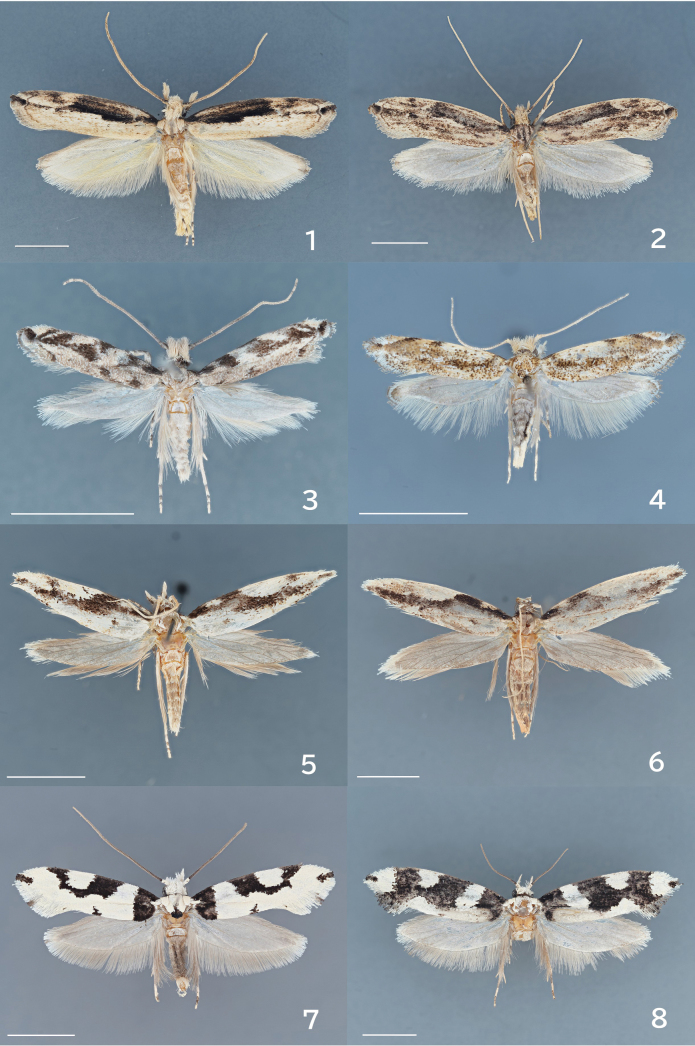
Adults. 1. *Erechthias itoi* Moriuti & Kadohara, 1994, male, museum ID ELKU-I-L-000181; 2. Ditto, female, ELKU-I-L-000182; 3. *E. zebrina* (Butler, 1881), male, genitalia slide no. JP-328; 4. *E. minuscula* (Walsingham, 1897), female, JP-315; 5. *E. atririvis* (Meyrick, 1931), male, JP-305; 6. Ditto, female, JP-306; 7. *E. mirabilis* sp. nov., male, holotype, JP-312; 8. Ditto, female, paratype, JP-018. Scale bars: 8.0 mm.

#### Diagnosis.

This species is externally similar to *E. atririvis* (Meyrick, 1931) and *E. charadrota* Meyrick, 1880 but can be distinguished by the following characteristics: the anterior 1/2 of the forewing is black from the base to basal 2/3 (the basal 2/5 is black with a white spot in *E. atririvis*, the black line is divided by a white line at the middle in *E. charadrota*). The male genitalia are similar to those of *E. chasmatias* (Meyrick, 1880), but can be distinguished by the phallus: the phallus with a cluster of ~15 distinct cornuti (phallus with large curved cornuti in *E. chasmatias*). The female genitalia are similar to those of *E. trigonosema* (Turner, 1923) but can be distinguished by the following characteristics: the ovipositor is relatively short, 2 × the length of segment VIII, and basal 1/5 of the ductus bursae is sclerotized (the ovipositor is relatively long, 4 × the length of segment VIII, and the ductus bursae is completely sclerotized in *E. trigonosema*).

#### Additional description.

***Measurements*.** Forewing length 6.5–8.2 mm (*n* = 5), antenna length 6.2–7.6 (*n* = 4) in male, forewing length 8.0–9.7 (*n* = 5), antenna length 7.1–7.7 (*n* = 3) in female. For more morphological information, see Moriuti and Kadohara (1994) and Yoshimatsu and Makihara (1995).

#### Distribution.

Japan (Chichijima Is., Hahajima Is., Iwoto Is.).

#### Biology.

The larvae feed on dead branches of *Cinnamomum japonicum* Siebold (Lauraceae) (Yoshimatsu and Makihara 1995). Adults have been collected almost every month, except for February, August, October, and December, on the Ogasawara Islands (Moriuti and Kadohara 1994).

#### DNA analyses.

The DNA barcode of a single specimen collected from Hahajima Island was completely matched to unidentified Arthropoda from British Indian Ocean Territory (Sequence ID: BIOT386-24). The intraspecific pairwise distance of this species among Ogasawara Island populations was 0.00%–1.86% (*n* = 12) (Suppl. material 2). The haplotype network was calculated and eight haplotypes were identified in 15 samples from Ogasawara Islands and Indo Ocean (Fig. 51).

#### Remarks.

Most specimens were collected using a light trap. Rarely seen during the daytime. This species is considered endemic to the Ogasawara Islands, but, unexpectedly, it is currently collected only on inhabited islands. In addition, as the DNA barcode of this species was matched to an unknown Arthropoda from British Indian Ocean Territory, this species may also inhabit areas other than the Ogasawara Islands.

### ﻿ Erechthias zebrina

Taxon classificationAnimaliaLepidopteraTineidae

(Butler, 1881)

1E0057CA-220F-5D04-9980-F9512694178B

Figs 3 , 28 , 36 , 45 , 51 , 52 Japanese name: Shima-chibi-tsumaorega

Argyresthia zebrina Butler, 1881: 403. Type locality: Hawaii (Honolulu).
Ereunetis zebrina : Walsingham 1907: 715, pl. 25, fig. 16.
Erechthias zebrina : Meyrick 1915b: 253; Fletcher 1921: 178; Meyrick 1928: 505; Meyrick 1930: 322; Viette 1949: 316; Clarke 1971: 196, figs 152, 153a-c, pl. 27, fig. h; Zimmerman 1978: figs 192, 194, 195; Robinson 1983: 307; Clarke 1986: 370, figs 258a-c, 316h; Robinson and Nielsen 1993: 310, fig. 650; Sakai 2013: 130, fig. 3-12-18; Shimizu 2020: 101, fig. 1; Nasu and Tamura 2020: 531, figs 1–8; Hirowatari and Yagi 2023: 24.
Ereunetis lanceolata Walsingham, 1897: 158. Type locality: Danish West Indies (St. Thomas). Synonymized by Meyrick 1915b: 253.
Ereunetis xenica Meyrick, 1911: 301. Type locality: Seychelles. Synonymized by Meyrick 1915b: 253.
Erechthias caustophara Turner, 1923: 186. Type locality: Australia. Synonymized by Robinson and Nielsen 1993: 310.
Tinexotaxa travestita Gozmany, 1968: 306, figs 8–11. Type locality: Siella Leon. Synonymized by Robinson 1983: 307; Hodges et al. 1983: 5.

#### Material examined.

**Japan: [Tokyo, Ogasawara Isls.]: [Chichijima Is.**]: 1♂, Higashimachi, alt. 6 m, 20. VI. 2022, SW, S. Yagi leg., genitalia slide no. JP-308, DNA sample JHP-270, museum ID ELKU-I-L-Bonin 000082, ELKU • 1♂, same locality, 12–14. III. 2023, T. Hirowatari & S. Yagi & M. Kimura & Y. Matsui & J.-H. Park leg., ELKU • 1♂, same locality, 11–12. VI. 2023, J.-H. Park leg., ELKU • 1♂, same locality, 13–14. VII. 2024, J.-H. Park leg., ELKU • 1♀, same locality, 17. VII. 2024, Y. Kawai leg., ELKU • 2♂, Ohgiura-Komagari, 18–31. VII. 2023, N. Tsuji leg., JP-328, ELKU • 1♂, Okumura, 2023. IX. 25, N. Tsuji leg., ELKU • [**Hahajima Is.**]: 1♀, Hahajima primary school, 20. IV. 1999, S. Omura leg., JP-304, ELKU • 1♂, Ruins of searchlight base, alt. 131 m, 16. III. 2023, LT, T. Hirowatari &. S. Yagi & M. Kimura & S. Tomura & Y. Matsui & J.-H. Park leg., ELKU.

#### Diagnosis.

This species is externally similar to *E. polionota* Turner, 1923 and *E. phileris* (Meyrick, 1893), but it can be distinguished by its forewing pattern, the costal margin has five cream lines at basal 1/8, 1/3, 2/3, subapical area, and apex in *E. zebrina* (the costal margin has four white to cream lines in *E. polionota* and *E. phileris*). The male genitalia of *E. zebrina* are also similar to those of *E. cyanosticta* (Lower, 1916) but can be distinguished by the slender uncus and elongated saccus (the uncus is broader, and the saccus is short and tongue-shaped in *E. cyanosticta*). The female genitalia are also similar to those of *E. darwini* Robinson, 1983 and *E. minuscula* but can be distinguished by a relatively large and broad signum (large but not curved in *E. darwini*, clearly small in *E. minuscula*).

#### Additional description.

***Measurements*.** Forewing length 3.5–4.1 mm (*n* = 6) in males, 3.9 mm (*n* = 1) in female. Antenna length 2.9–3.6 mm (*n* = 3) in male. For more morphological information, see Clarke (1971) and Zimmerman (1978).

#### Distribution.

Japan (Honshu, Yakushima Is., Amamiohshima Is., Ishigakijima Is., Chichijima Is., Hahajima Is.); Pantropical: China, Ceylon (Sri Lanka), India, Java, Sumatra, Malaysia, Bali, Borneo, Hawaii, Rapa, Fiji, Society islands, Marquesas Islands, Caroline Islands, Australia, Mexico, Saint Thomas, Puerto Rico, Panama Canal Zone, Cuba, Jamaica, Brazil, Sierra Leone, Congo, Cameroun, Madagascar, Seychelles, Mauritius (Zimmerman 1978; Clarke 1986; Robinson and Nielsen 1993; Robinson et al. 1994; Sakai 2013; Shimizu 2020).

#### Biology.

Larvae feed on large amounts of detritus, fruits of *Cola acuminata* (P. Beauv.) Schott & Endl. (Malvaceae), false cotton, galls of *Lophira alata* Banks ex Gaertn (Ochnaceae), and dry or decaying vegetable matter (Clarke 1986). Larvae of this species also feed on bat guano in Okinawa (Nasu and Tamura 2020). Adults were collected from Ogasawara Islands in March, April, June, and July.

#### DNA analyses.

The DNA barcode of a single specimen of *E. zebrina* was matched to *E. zebrina* (Butler, 1881) from Costa Rica (Sequence ID: LTOLB433-09), based on the identification engine of BOLD Systems, and the similarity between them was 100%.

#### Remarks.

This species is common on the streets of residential areas on Chichijima Island.

### ﻿ Erechthias minuscula

Taxon classificationAnimaliaLepidopteraTineidae

(Walsingham, 1897)

921A8CE7-6382-5658-B11E-A9E18013658C

Figs 4 , 29 , 37 , 46 , 51 , 52 Japanese name: Nanyo-hime-tsumaorega

Ereunetis minuscula Walsingham, 1897: 155. Type locality: West Indies (St. Thomas); Walsingham 1907: 716; Swezey 1909: 12; Swezey 1912: 155; Walsingham 1914: 347; Wolcott 1923: 205; Swezey 1929: 281; Forbes 1930: 147; Wolcott 1936: 501; Swezey 1940: 458; Wolcott 1948: 739; Beardsley 1961: 354.
Decadarchis minuscula : Meyrick 1929: 505; Harris 1937: 486; Ghesquière 1940: 86; Vesey-Fitzgerald 1941: 158; Lepesme 1947: 318; Viette 1949: 316; Swezey 1942: 215; Davis 1953: 85; Swezey 1952: 378; Diakonoff [1968]: 265, 308; Clarke 1971: 211; Clarke 1986: 361; Zimmermann 1978: 352.
Lepidobregma minuscula : Zimmermann 1978: 352; Davis 1983: 5; Robinson and Nielsen 1993: 289.
Erechthias minuscula : Davis 1984: 21; Robinson and Kirke 1990: 133; Robinson 2009: 20–25, 51, fig. 36; Robinson and Nielsen 1993: 295, 310; Heppner 2003: 236; Hirowatari et al. 2012: 107, figs 3E, 4B; Cock and Burris 2013: 15, figs 2g, 7; Hirowatari and Yagi 2023: 24.

#### Material examined.

**Japan: [Tokyo, Ogasawara Isls.]: [Nakodojima Is.**]: 1♀, 16. VII. 2024, SW, J.-H. Park leg., DNA sample JHP-202, ELKU, 1♀, same locality, Host coll. on 16. VII. 2024, Emrg. on 2024. VIII. 8, Host: decaying wood of *Pandanus boninensis* Warb. (1900) with white fungi, J.-H. Park leg., ELKU; 1♀, same data but Emrg. on 19. VIII. 2024, ELKU • [**Chichijima Is.**]: 1♂, Asahiyama, 13. XI. 2022, T. Hirowatari leg., ELKU • 1♂, Shigureyama, alt. 252 m, 18. VI. 2022, LT, S. Yagi, T. Hirowatari, S. Tomura, M. Kimura leg., genitalia slide no. JP-309, JHP-271, museum ID ELKU-I-L-Bonin 000080, ELKU • 1♂, Ohmura-beach, alt. 0 m, Larvae coll. on 27. VI. 2022, Emrg. on 6. VII. 2022, Host: *Sophora tomentosa* mature legume, JHP-272, ELKU-I-L-Bonin 000081, ELKU • 1♂, same data, but Emrg. on 29. VII. 2022, ELKU • 1♂, same data, but Emrg. on 8. VIII. 2022, ELKU • 1♂, same data, but Emrg. on 16. VIII. 2022, ELKU • 1♂, same data, but Emrg. on 9. IX. 2022, ELKU • 2♂1♀, Ogamiyama Park, 13. VI. 2023, LT, J.-H. Park leg., ELKU • 1♀, Higashimachi, 13–14. VII. 2024, J.-H. Park leg., ELKU • [**Hahajima Is.**]: 1♀, Shin yûhigaoka, 14. VI. 2023, LT, J.-H. Park leg., JP-307, JHP-201, ELKU • 1♀, same data, JP-315, ELKU.

#### Diagnosis.

This species is externally similar to *E. iolaxa* Moriuti & Kadohara, 1994, but it can be distinguished by the following characteristics: the costal margin has two brown lines in the forewing (absent in *E. iolaxa*); the apex of the valva is rounded in the male genitalia (sharp in *E. iolaxa*); the ductus bursae are twisted; and the signum is simply knob-shaped in the female genitalia (relatively large and curved in *E. iolaxa*).

#### Additional description.

***Measurements*.** Forewing length 3.9–4.4 mm (*n* = 3), antenna length 3.2, 3.3 mm (*n* = 2) in male, forewing length 4.2–5.2 mm (*n* = 5), antenna length 3.3–3.7 mm (*n* = 3) in female. For more morphological information, see Davis and Mendel (2013).

#### Distribution.

Japan (Minamidaitohjima Is., Nakodojima Is., Chichijima Is., Hahajima Is.), Pantropical, Australia, and South America (Clarke 1986; Hirowatari et al. 2012; Davis and Mendel 2013).

#### Biology.

The larvae feed on various dead plant materials (Zimmerman 1978). This species emerged from nests of the Bull-headed Shrike, *Lanius bucephalus* Temminck & Schlegel, 1847 (Laniidae), on Minamidaitojima Island, Japan (Hirowatari et al. 2012). On the Ogasawara Islands, larvae emerged from mature legumes of *Sophora tomentosa* L. (Fabaceae). Adults were collected from Ogasawara Islands in June, July, and November.

#### DNA analyses.

The DNA barcode of this specimen is matched to *E. minuscula* (Walsingham, 1897) from Trinidad and Tobago (FRUT734-14) based on the identification engine of BOLD Systems, and the similarity between them was 99.85%. The intraspecific pairwise distance of this species among Ogasawara Island populations was 0.00% (*n* = 4) (Suppl. material 2).

#### Remarks.

Two adults of this species emerged from the decaying wood of *Pandanus boninensis* (Pandanaceae) with white fungi (Polyporaceae) obtained from Nakodojima Island, along with *Morophaga formosana* Robinson, 1986 (Tineidae, Scardiinae).

### ﻿ Erechthias atririvis

Taxon classificationAnimaliaLepidopteraTineidae

(Meyrick, 1931)

C9EE2345-F1BB-5A9C-8B87-C9E1D5CEC642

Figs 5 , 6 , 30 , 38 , 52 Japanese name: kurosuji-tsumaorega

Decadarchis atriviris Meyrick, 1931:166. Type locality: Japan (Tokyo); Issiki 1950: 443, fig. 1191; Issiki 1957: 15, pl. 2, fig. 52; Okano 1959: 275, pl. 182, fig. 18; Moriuti 1982: pl. 2, figs 23, 24, pl. 237, fig. 1; Park 1983: 56; Azuma and Kinjo 1987: 74; Yoshimatsu and Sakamoto 1991: 99, figs 1–5; Yoshimatsu 1992: 779, figs 1–4, 6, 14.
Erechthais atririvis : Davis 1992: 64; Moriuti and Kadohara 1994: 569; Lee et al. 2020: 625, figs 1A, 2A, 3A; Sakai 2013: 130, fig. 3-12-15; Hirowatari and Yagi 2023: 24; Park et al. 2024b: 1051, fig. 5.

#### Material examined.

**Japan: [Tokyo, Ogasawara Isls.]: [Hahajima Is.**]: 1♂1♀, 1–11. V. 1977, M. Takakuwa, from Ogsawara Islands, Hahajima Island, rotten Shimayabunikkei, (collected in VII. 1976), Yokohama (1~11. V. 1977), genitalia slide no. JP-305 (♂), JP-306 (♀), NMNS.

#### Diagnosis.

This species is externally similar to whitish *Erechthias* species, *E. itoi*, *E. acrodina* Meyrick, 1912 and *E. charadrota* but it can be distinguished by the following characteristics: from the base to the basal 1/3 of the costal margin is black, but the middle is interrupted by a cream to white spot in the forewing (almost black, but with a narrow white line in *E. itoi*; from the base to the basal 1/4 cream without black scales in *E. acrodina*; completely black in *E. charadrota*). The male genitalia are also similar to those of *E. itoi*, *E. acrodina*, and *E. charadrota*, but it can be distinguished by the shape of valva: the shape of the valva is oval, without the basicostal process, and the ventral margin is slightly rounded (the elongate basicostal process is present and the ventral margin curved at apical 1/3 in *E. itoi*, the basicostal process is absent and slightly concaved at ventral margin in *E. acrodina*, the shape of valva rounded is rectangle and the basicostal process is absent in *E. charadrota*). The female genitalia are also similar to those of *E. itoi*, but it can be distinguished by the shape of signum: the signum is small, simple knob-shaped, and with no additional lobes (the signum is absent in *E. itoi*).

#### Additional description.

***Measurements*.** Forewing length 6.8 mm in male, 8.1 mm in female, antenna length 4.8 mm in male, 5.0 mm in female in Ogasawara Islands. Wingspan 13 mm in male in original description.

#### Distribution.

Japan (Honshu, Shikoku, Kyushu, Tsushima Is., Hahajima Is., Okinawajima Is., Ishigakijima Is., Iriomotejima Is., Yonagunijima Is.), Taiwan, and South Korea (Sakai 2013; Lee et al. 2020; Park et al. 2024b).

#### Biology.

Larvae bore under the rotten bark and feed on the cambium. Adults were collected in June and July in Honshu and from May to July on Ishigaki Island (Sakai 2013). On the Ogasawara Islands, two adults emerged from rotten wood (see remarks) in May.

#### DNA analyses.

DNA barcodes were not generated.

#### Remarks.

Since there is no plant called “Shima-yabunikkei,” as was labeled by M. Takakuwa, it is assumed that the name refers to the *Cinnamomum pseudopedunculatum* Hayata (Ogasawara-yabunikkei). This species was not collected during the study period (2022–2024).

### ﻿ Erechthias mirabilis sp. nov.

Taxon classificationAnimaliaLepidopteraTineidae

8150D0A4-4D54-55DF-9434-88C26C6D8876

https://zoobank.org/1DBE12C6-5F18-49D8-A458-6EF442C3328F

Figs 7 , 8 , 17 , 18 , 31 , 39 , 47 , 51 , 52 Japanese name: Chichijima tsumaorega

#### Type material.

***Holotype*: Japan**: ♂, Tokyo Met., Ogasawara Isls., Chichijima Is., Kitafukurozawa, alt. 10 m, LT, 26. IX. 2023, Y. Matsui leg., genitalia slide no. JP-312, DNA sample JHP-200, ELKU. ***Paratypes*: Japan: [Tokyo, Ogasawara Isls.]: [Chichijima Is.**]: 1♀, 16–17. VII. 2002, K. Oyama leg., ELKU 1♂, Chichi-jima Is., Kiyose, 27. IX. 2020, M. Kimura leg. • 1♀, same data, genitalia slide no. JP-321, DNA sample JHP-274, Museum ID ELKU-I-L-Bonin 000104, ELKU • 1♀, Ogamiyama, 25. VI. 2022, S. Yagi & S. Tomura leg., genitalia slide no. JP-018, DNA sample JHP-273, Museum ID ELKU-I-L-Bonin 000066, ELKU • 1♀, Ohgiura, VI. 2023, N. Tsuji leg., ELKU • 1♂, Higashimachi, LT, 23. XII. 2023, Yu Hisasue leg., JP-326, JHP-153, ELKU • 1♂, Komagari, 2024. II. 4, N. Tsuji leg., EKKU • 1♀, Okumura, 2024. VIII. 19, N. Tsuji leg., EKKU • [**Anijima Is.**]: 1♂, Mt. Maru-yama, 14. XI. 2022, T. Hirowatari, M. Kimura, & J.-H. Park leg., ELKU.

#### Diagnosis.

The new species is externally similar to *Erechthias simulans* (Butler, 1882) from the tropical Pacific and *E. dissimulans* (Meyrick, 1915c) from Sri Lanka, but can be distinguished by the following characteristics: the basal area of the forewing is fully black in *E. mirabilis* sp. nov. (only black spots in *E. simulans* and *E. dissimulans*). The male and female genitalia are also similar to those of *E. simulans* but they can be distinguished by the following characteristics: the valva is broad, oval, and slightly concave at the costal margin in the male genitalia (the valva is rounded, dagger-shaped, and bulged at the middle of the costal margin in *E. simulans*); the ostium is smaller and weakly concave; and the anterior 1/2 of the signum is sharp and curved in the female genitalia (curved but rounded in *E. simulans*).

#### Description.

**Adults. Male.** (Figs 7, 17) Forewing length 6.8 mm, antenna length 6.0 mm in holotype. Forewing length 6.7, 7.8 mm (*n* = 2), antenna length 5.0, 5.7 mm (*n* = 2) in paratypes. ***Head*.** Frons and vertex whitish cream. Labial palpus whitish cream, first palpomere covered with black scales; second palpomere with robust brush hairs, outer surface black, with a few black bristles. Antenna filiform, notch present at base; scape whitish cream; flagellum black, gradually whiter toward tip. ***Thorax*.** Anterior 3/4 of mesonotum whitish cream, posterior 1/4 black. Tegula whitish cream, basally black. Foreleg black to dark gray, outer surface of tibia and basal 1/2 of tarsus cream white. Midleg black to dark gray, each tarsomere with cream band at distal end. Hindleg fuscous gray, tibia bearing with long hairs, basal 1/3 and subapical aera of tarsus black. Forewing venation with Sc, R1–3 and R4+5, M1 and M2+3, CuA1–2 and CuP, 1A+2A present; R1 from basal 3/7 of discal cell to apical 1/3 of costa; 1A fused with 2A from basal 2/5 of wing to end; chorda weak. Apex of forewing weakly upturned. Forewing ground color white, with black pattern; basal 1/6 black; middle of anterior 1/2 with a large irregularly U-shaped spot; apex with a rectangular black spot; middle of dorsum with a small wedge-shaped black spot; tornus with one black scale; cilia white, middle of marginal area with a gray spot. Hindwing venation with Sc+R1, Rs, M1 and M2+3 CuA1–2 and CuP, 1A+2A present; 1A+2A strongly angled; frenulum a single slender bristle. Hindwing and cilia fuscous cream. ***Abdomen*.** Covered with fuscous cream scales.

**Female.** (Figs 8, 18, 47) Forewing length 6.5–7.4 mm (*n* = 4), antenna length 5.4 mm (*n* = 1) in paratypes. Almost same as male except for antenna and forewing; notch absent in antenna; apex of forewing slightly upturned; chorda well-developed; forewing ground color black, with four white spots: basal 1/5, apical 1/3 of costa, tornus, and apex.

**Male genitalia.** (Fig. 31) Uncus thick, short, a pair of membranous lobes, apical corner with a few bristles. Tegumen strongly sclerotized, fused with vinculum, formed into broad ring. Vinculum robust, ~1.5 × length of tegumen; saccus broad triangular. Valva broad oval, roughly covered with bristles; costa broad with short, dense spines, basicostal process absent; basal 2/3 of ventral margin almost straight, gently curved toward apex. Juxta a strongly sclerotized pouch. Phallus cylindrical, almost same length of valva; vesica with a robust needle-like cornutus and many small spicular cornuti.

**Female genitalia.** (Fig. 39) Ovipositor 2.5 × length of segment VII, papillae analis with short bristles; apophysis anterioris thick, ~1/2 length of apophysis posterioris. Segment VIII long, posterior margin with ~15 bristles; tergum VIII with stout dorsal rami fusing with apophysis anterioris; ostium elongated piriform with dense small spines, located at anterior 1/2 of sternum VIII. Ductus bursae tubular, 1.4 × length of corpus bursae, anterior 1/6 broad, wrinkled and curved toward corpus bursae. Corpus bursae oval; signum large flatten claw-shaped with robust rounded rectangular projection.

#### Distribution.

Japan (Chichijima Is., Anijima Is.).

#### Biology.

Host is unknown. Adults have been collected in June, July, and September in Chichijima Island.

#### Etymology.

The name of this species is derived from the Latin *mirabilis*, because this species is the most marvelous *Erechthias* to occur in Japan.

#### DNA analyses.

The DNA barcodes of this species were most similar to *Erechthias* sp. ANIC1 from Australia (ANICI492-10) based on the identification engine of BOLD systems, and the similarity between them was 92.51%. These DNA barcodes were also similar to *Erechthias simulans* from Moorea Island in the Society Islands (French Polynesia) (PMANL5097-16), with similarity of 90.67%. The intraspecific pairwise distance of this species was 0.00% (*n* = 3) (Suppl. material 2).

#### Remarks.

This species is endemic to the Chichijima Islands. The most similar species, *E. simulans* is widely spread in the tropical Pacific: Hawaii, Solomon Islands, Fiji, Samoa, Elice Islands, Midway, Society Islands, Marquesas Islands, and Australia (Clarke 1986). This new species may have originated in the Pacific Ocean and Australia.

### ﻿ Erechthias nidumicola sp. nov.

Taxon classificationAnimaliaLepidopteraTineidae

1C25F718-E853-5C4C-A2AB-23B625190FA2

https://zoobank.org/99281F98-7E9C-4810-8148-CD3FD885D099

Figs 9 , 10 , 19 , 20 , 32 , 40 , 48 , 49 , 51 , 52 Japanese name: Torinosu-tsumaorega

Erechthias sp. Nasu et al. 2014: 76, 75, fig. 12; Hirowatari and Yagi 2023: 24.

#### Type material.

***Holotype*: Japan**: ♂, Tokyo Met., Ogasawara Isls., Chichijima Is., Kopepe beach, 14. III. 2023, T. Hirowatari, S. Yagi, M. Kimura, Y. Matsui, J.-H. Park leg., genitalia slide no. JP-310, DNA sample JHP-088, ELKU. ***Paratypes*: Japan: [Tokyo, Ogasawara Isls.]: [Mukojima Is.**]: 1♂2♀, 15. VII. 2024, SW, J.-H. Park leg., genitalia slide no. JP-373(♂), DNA sample JHP-213(♂), ELKU • 1♂, same data, JHP-207, ELKU •1♀, same locality, 15. VII. 2024, LT, S. Yagi leg., ELKU • [**Nakodojima Is.**]: 6♂, 16. VII. 2024, S. Yagi leg., JHP-214, ELKU • [**Ototojima Is.**]: 2♂, 12–13. VII. 2024, J.-H. Park leg., JHP-210, ELKU • 1♀, Kurohama, 12–13. VII. 2024, LTFIT, M. Kimura leg., ELKU • [**Anijima Is.**]: 1♀, alt. 162 m, 20. VI. 2022, S. Tomura leg., Beating: dead leaves of *Pandanus boninensis*, ELKU • 1♂1♀, North of C Line, 11. III. 2023, T. Hirowatari, S. Yagi, M. Kimura, S. Tomura, Y. Matsui & J.-H. Park leg., genitalia slide no. JP-174 (♂), JHP-118 (♂), ELKU • [**Nishijima Is.**]: 1♂, em. 9. VIII. 2013, 13–43, Host: nest of *Puffinus pacificus*, Y. Nasu leg., genitalia slide no. YN 1540, OMU • 1♀, same data, em. 12. VIII. 2013, genitalia slide no. YN 1541, OMU • [**Chichijima Is.**]: 2♂, Kopepe beach, 14. III. 2023, T. Hirowatari leg., ELKU • 1♂, same locality, 14. VII. 2024, T. Hirowatari leg., ELKU • 1♀, Higashi-machi, 12–14. III. 2023, T. Hirowatari, S. Yagi, M. Kimura, Y. Matsui & J.-H. Park leg., JP-140, JHP-349, ELKU • 1♀, Mt. Mikazuki-yama, SW, 13. VI. 2023, J.-H. Park leg., JP-141, JHP-092, ELKU • 1♂, Nagasaki observatory, 14. III. 2023, T. Hirowatari, S. Yagi, M. Kimura, Y. Matsui & J.-H. Park leg., ELKU • 1♂, Kominato–Nakayama-toge, 10. III. 2023, T. Hirowatari leg., ELKU • 1♂, Asahiyama, 11. VI. 2023, T. Hirowatari leg., ELKU • 1♀, Chuosan, 11. VII. 2024, J.-H. Park leg., ELKU • [**Hahajima Is.**]: 2♀, Minamizaki, 10–11. XI. 2022, SW, J.-H. Park leg., JP-323, ELKU • 2♂1♀, same data, T. Hirowatari leg., ELKU • 1♂2♀, same locality, 15. III. 2023, T. Hirowatari, S. Yagi, M. Kimura, Y. Matsui, N. Katsube & J.-H. Park leg., ELKU • 1♂, same data, JP-142, JHP-093, ELKU • 2♂, same data, S. Yagi leg., ELKU • 2♂, same data, T. Hirowatari leg., ELKU • 1♂, same locality, Host coll. on 15. III. 2023, Emrg. on 6. V. 2023, Host: brown fungi, J.-H. Park leg., JP-129, JHP-084, ELKU • 1♀, same locality, coll. on 15. III. 2023, Emrg. on 31. III. 2023, J.-H. Park leg., JP-132, JHP-087, ELKU • 1♂1♀, same locality, 14. VI. 2023, T. Hirowatari leg., ELKU • 1♀, same data, museum ID ELKU-IL Bonin 000183, ELKU • 1♂, Funakiyama, 24. VI. 2022, S. Tomura leg., Beating: dead leaves of Livistona chinensis var. boninensis or *Pandanus boninensis*, JP-178, JHP-348, ELKU • 1♂, Funamidai, 21. VI. 2022, S. Tomura leg., Beating: dead leaves of Livistona chinensis var. boninensis or *Pandanus boninensis*, ELKU • 1♀, Chokiyama, alt. 196 m, 22. VI. 2022, LT, S. Yagi, T. Hirowatari, S. Tomura & M. Kimura leg., JHP-280, ELKU-I-L-Bonin 00075, ELKU • [**Mukohjima Is.**]: 1♀, 15. VI. 2023, daytime search, Y. Matsui leg., ELKU • 2♀, same data, SW, S. Yagi leg., ELKU • 1♂, same data, T. Hirowatari leg., JHP-106, ELKU • 1♀, same locality, 16. VII. 2024, Y. Matsui leg., ELKU • [**Hirajima Is.**]: 3♂, alt. 20 m, 23. IX. 2023, daytime search, Y. Matsui leg., ELKU • 1♂, same data, JP-371, JHP-211, ELKU • [**Meijima Is.**]: 1♂, 16. VI. 2023, daytime search, Y. Matsui leg., ELKU • 1♂1♀, same data, S. Yagi leg., SW, JHP-212(♂), ELKU.

**Figures 9–16.**
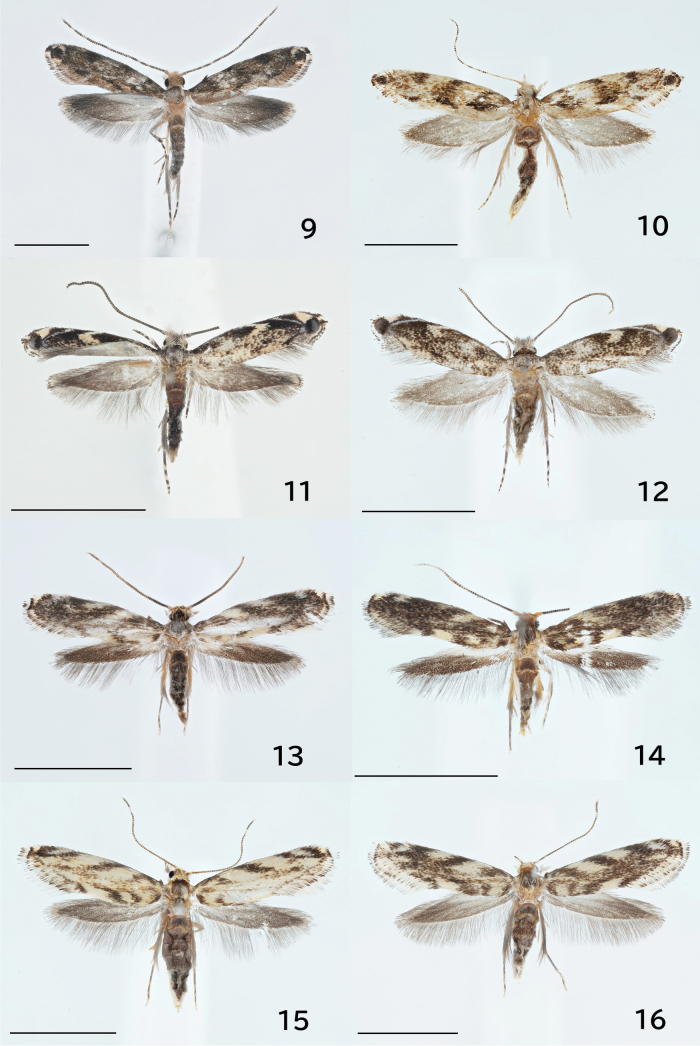
Adults. 9. *Erechthais nidumicola* sp. nov., male, holotype, JP-310; 10. Ditto, female, paratype, museum ID ELKU-I-L-000183; 11. *E. oculus* sp. nov., male, holotype, genitalia slide no. JP-128; 12. Ditto, female, paratype, ELKU-I-L-000184; 13. *E. flavimacula* sp. nov., male, holotype, JP-325; 14. Ditto, male, paratype, DNA sample JHP-206; 15. Ditto, female, paratype, ELKU-I-L-000185; 16. Ditto, female, paratype, ELKU-I-L-000186. Scale bars: 8.0 mm.

**Figures 17–24.**
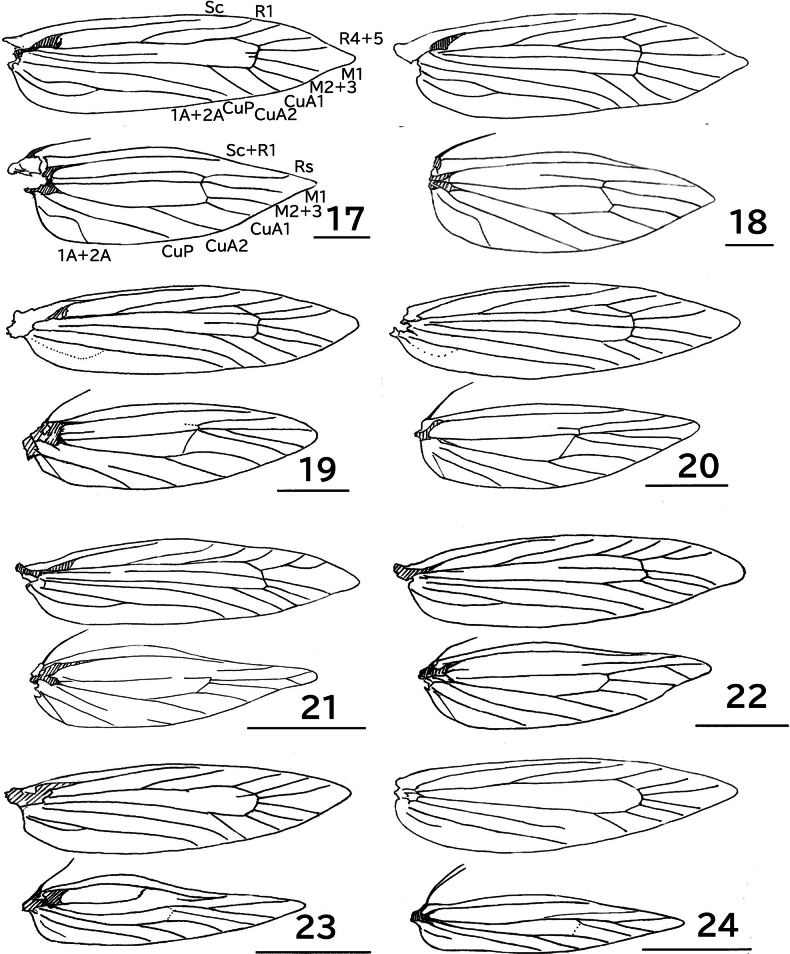
Wing venations. 17. *Erechthias mirabilis* sp. nov., male; 18. Ditto, female; 19. *E. nidumicola* sp. nov., male; 20. Ditto, female; 21. *E. oculus* sp. nov., male; 22. Ditto, female; 23. *E. flavimacula* sp. nov., male; 24. Ditto, female. Scale bars: 1.0 mm.

#### Diagnosis.

The new species is similar to *E. aspera* (Clarke, 1986), *E. celestra* (Clarke, 1986), and *E. incongrua* (Clarke, 1986), but can be distinguished by the following characteristics: the forewing has three black to brown spots at the costal margin, and the ground color is ocherous and clearly paler than that of other species in females; the valva is broad and the saccus is short in the male genitalia (the valva small and the saccus is relatively long in other related species). The female genitalia of this species are also similar to those of *E. trigonosema*, but it can be distinguished by the following characteristics: the ductus bursae lacks sclerotization, the corpus bursae is weakly sclerotized at the posterior 1/5 (the ductus bursae sclerotized and with dense small spines, and the corpus bursae is weakly sclerotized at the posterior 2/5 in *E. trigonosema*).

#### Description.

**Adults. Male.** (Figs 9, 19, 48) Forewing length 5.5 mm, antenna length 5.0 mm in holotype. Forewing length 4.2–5.8 mm (*n* = 9), antenna length 3.9–4.4 (*n* = 4) in paratypes. ***Head*.** Vertex ocherous white, frons cream. Labial palpus cream; second palpomere with robust brush, outer surface dark brown, with few black bristles; Antenna filiform, brown, notch absent; flagellum with seven or eight cream bands. ***Thorax*.** Mesonotum and tegula dark brown. Foreleg dark gray, inner surface ocherous gray. Midleg cream; outer surface of tibia, and basal 1/3, middle, and subapical area of tarsus dark gray. Hindleg cream, outer surface dark gray; tibia bearing with long hairs; distal end of each tarsomere with cream ring. Forewing venation with Sc, R 1–3 and R4+5, M1 and M2+3, CuA1–2 and CuP, 1A+2A present; R1 from basal 1/3 of discal cell to apical 2/5 of costa; 1A fused with 2A from basal 1/3 to end; chorda well developed. Apex of forewing upturned. Forewing ground color brown, with black and ocherous white spots; costal margin with three trapezoidal black spots at base, basal 1/3 and 2/3; tornus with a small narrow ocherous white spot; apex with a black dot bordered by ocherous white lines on the front and back and formed into eye spot; cilia black at apex, cream at subapical area; outer margin fuscous brown. Hindwing venation with Sc+R1, Rs, M1–3, CuA1–2 and CuP, 1A+2A, 3A present; M1 and M2 stalked at basal 1/5 of wing; frenulum a single slender bristle. Hindwing gray, cilia gray. ***Abdomen*.** Covered with gray scales.

**Female.** (Figs 10, 20, 49) Forewing length 4.7–5.7 (*n* = 6) antenna length 3.3–3.8 mm (*n* = 3) in paratypes. Almost same as male but body ground color relatively light; head ocherous white, frons cream; thorax and tegula ocherous white, brown basally; forewing ground color ocherous, with dark brown spots.

**Male genitalia.** (Fig. 32) Uncus broad, a pair of membranous lobes, apical area with a few bristles. Tegumen fused with vinculum, formed into ring. Vinculum ~2 × length of tegumen; saccus narrow, semicircular. Valva broad blade-shaped; ventral margin gently curved toward apex with long bristles; basicostal process absent, costa convex, with short dense spines. Juxta is a strongly sclerotized pouch. Phallus thick, cylindrical, weakly curved, 1.2 × length of valva; vesica with one pair of claw-shaped cornuti and with many small spicular cornuti.

**Female genitalia.** (Fig. 40) Ovipositor 2.5 × length of segment VIII; papillae analis sharp, with a few bristles; apophysis anterioris relatively thick, 0.7 × length of apophysis posterioris. Segment VIII broad rectangular, posterior margin with ~20 bristles; tergum VIII with dorsal rami fused with apophysis anterioris; ostium sclerotized funnel-shaped with dense small spines, located at anterior margin of sternum VIII. Ductus bursae tubular, 1.5 × length of corpus bursae, gently broad toward corpus bursae. Corpus bursae oval, posterior 1/4 slightly twisted and sclerotized; signum absent.

#### Distribution.

Japan (Mukojima Is., Nakodojima Is., Ototojima Is., Anijima Is., Nishijima Is., Chichijima Is., Hahajima Is., Mukohjima Is., Meijima Is., Hirajima Is.).

#### Biology.

One specimen has emerged from *Ganoderma orbiforme* (Fr.) Ryvarden (2000) (Polyporales: Ganodermataceae). This species has also been obtained from nests of *Puffinus pacificus* (Gmelin, 1789) (Nasu et al. 2014). Adults have been collected from Ogasawara Islands in March, June, July, September, and November.

#### Etymology.

The name of the new species is derived from the Latin *nidum* (nests) + *incola* (to inhabit), referring to where this species was first discovered, i.e., in the nest of *Puffinus pacificus*.

#### DNA analyses

(Fig. 51, Suppl. material 2). The intraspecific pairwise distances of this species were 0.46%–4.71% (*n* = 13) (Suppl. material 2). Thirteen samples were used in this analysis, and all of them were unique haplotypes.

#### Remarks.

This species is endemic to Ogasawara Islands. During this study, many specimens of this species were collected by beating dead leaves of Livistona chinensis var. boninensis (Arecaceae) or *Pandanus boninensis* (Pandanaceae). This species is the same as *Erechthias* sp., which was recorded by Nasu et al. (2014) from a nest of *Puffinus pacificus*.

### ﻿ Erechthias oculus sp. nov.

Taxon classificationAnimaliaLepidopteraTineidae

A06ECA4A-BECD-5078-9501-2C3D6A9C874B

https://zoobank.org/42A31413-7963-4846-808D-7CF1FCC4B777

Figs 11 , 12 , 21 , 22 , 33 , 41 , 51 , 52 Japanese name: Medamamon-tsumaorega

#### Type material.

***Holotype*: Japan**: ♂, Tokyo Met., Ogasawara Isls., Hahajima Is., Minamisaki, SW, 10–11. XI. 2022, J.-H. Park leg., genitalia slide no. JP-128, DNA sample JHP-082, ELKU. ***Paratypes*: Japan: [Tokyo, Ogasawara Isls.]: [Anijima Is.**]: 1♀, alt. 162 m, 20. VI. 2022, Beating: dead leaves of *Pandanus boninensis*, S. Tomura leg., genitalia slide no. JP-133, DNA sample JHP-089, ELKU • [**Chichijima Is.**]: 1♀, Mikazukiyama, Host coll. on 13. VI. 2023, Emrg. on VII. 2023, Host: litter, J.-H. Park leg., genitalia slide no. JP-317, DNA sample JHP-203, ELKU • [**Hahajima Is.**]: 1♂, Kitakou, 9. XI. 2022, T. Hirowatari leg., JP-166, JHP-081, ELKU • 1♀, Minamisaki, 10–11. XI. 2022, T. Hirowatari leg., museum ID ELKU-IL Bonin 000184, ELKU • 1♂; same locality, 12. XI. 2022, T. Hirowatari leg., JP-322, ELKU • 1♂, Tamagawa-dam, 17. III. 2023, LT, T. Hirowatari, S. Yagi, M. Kimura, S. Tomura, Y. Matsui & J.-H. Park leg., ELKU.

#### Diagnosis.

The new species is similar to *E. polionota* Turner, 1923, *E. phileris* (Meyrick, 1893) and *E. zebrina* (Butler, 1881) but can be distinguished by the following characteristics: the forewing has a falciform cream spot at the middle of the costal margin. The genitalia are also similar to those of *E. zebrina*, but can be distinguished by the following characteristics: the valva is narrow tongue-shaped, the dorsal margin has a finger-shaped lobe in the male genitalia (the valva curved and additional lobe absent in *E. zebrina*), the signum is large and hook-shaped, and the posterior 1/3 has a twisted trapezoidal lobe (curved spatula, posterior side with small short rod-shaped lobe in *E. zebrina*) in the female genitalia.

#### Description.

**Adult. Male** (Figs 11, 21) Forewing length 3.5 mm, antenna length 2.7 mm in holotype. Forewing length 3.1–3.3 mm (*n* = 3) in paratypes. ***Head*.** Vertex fuscous brown, frons brown. Labial palpus cream, outer margin of second palpomere covered with dark brown scales, with a few black bristles. Antenna filiform, notch absent; scape fuscous ocherous, simple fusiform; pedicel and flagellomere dark gray. ***Thorax*.** Mesonotum and tegula black, apex of each scale white. Foreleg dark gray, inner surface ocherous; middle and distal end of tibia, and distal end of each tarsomere with cream bands. Midleg cream; proximal end, basal 1/3, 2/3 of tibia, and distal end of each tarsomere with cream bands. Hindleg cream, tibia bearing long ocherous gray hairs; distal end of each tarsomere with cream bands. Forewing venation with Sc, R1–5, M1 and M2+3, CuA1–2 and CuP, 1A+2A present; R1 from middle of discal cell to middle of costa; R4 and R5 stalked from middle 1A fused with 2A from basal 2/5 of wing to end. Apex of forewing weakly upturned. Forewing ground color black with cream and dark silver spots, posterior 1/2 scattered with cream scales; middle of costal margin with falciform cream spot; narrow silver line elongated from apical 1/3 of costa to eye spot; apical area with a silver and black eye spot, anteriorly with a large triangle, cream spot; cilia outer margin with two black line; underside cream; apex with small cream spot. Hindwing venation with Sc+R1, Rs, M1–3, CuA1–2 and CuP, 1A+2A, 3A present; M1 and M2 stalked at near apex; frenulum a single slender bristle. Hindwing fuscous gray, basal 1/4 of costa with ocherous scales; cilia fuscous gray. ***Abdomen*.** Covered with black scales.

**Female** (Figs 12, 22) Forewing length 4.1, 4.3 mm (*n* = 2), antenna length 3.3 mm (*n* = 1) in paratypes. Almost same as male but antenna relatively narrow, and hindwing without ocherous scales at base.

**Male genitalia.** (Fig. 33) Uncus simple, short, a pair of membranous lobes, apex with few bristles. Tegumen broad, fused with vinculum. Vinculum narrow, long; saccus slender, elongated triangle. Valva narrow, spatulate with rough hairs at ventral and costa; ventral margin almost straight; basicostal process elongated narrow finger-shaped, apex with short dense spines. Juxta strongly sclerotized, long trapezoid. Phallus narrow cylindrical, curved, 2.1 × length of valva; apex with one carina, comprising seven or eight small teeth; vesica with spicular cornuti.

**Female genitalia.** (Fig. 41) Ovipositor 4 × length of segment VIII; papillae analis sharp, roughly with short bristles; apophysis posterioris 1.5 × length of apophysis anterioris. Segment VIII rounded long trapezoidal, posterior margin slightly concave and with 8–10 setae; tergum VIII with dorsal rami connected to apophysis anterioris; ostium located at anterior margin of sternum VIII; antrum funnel-shaped, weakly sclerotized. Ductus bursae thick tubular, 1.5 × length of corpus bursae, anterior 3/4 sclerotized, gently broad toward corpus bursae. Corpus bursae irregularly oval; signum large sickle-shaped with a twisted trapezoidal lobe.

#### Distribution.

Japan (Anijima Is., Chichijima Is., Hahajima Is.).

#### Biology.

Larval host is unknown, but only one female emerged from litter. Adults have been collected in June and September on Ogasawara Islands.

#### Etymology.

The name of the new species is derived from the Latin *oculus* (eye), which refers to the presence of a large eye spot on the forewing. The eye spot of this species appears larger than those of other Japanese *Erechthias* species because of the presence of a cream triangular spot (resembling a sclera).

#### DNA analyses.

The intraspecific pairwise distances of this species were 0.00%–0.15% (*n* = 4) (Suppl. material 2).

#### Remarks.

This species is Endemic to the Ogasawara Islands:

### ﻿ Erechthias flavimacula sp. nov.

Taxon classificationAnimaliaLepidopteraTineidae

2D5D9752-6D3E-505D-BA69-44DFDA45D670

https://zoobank.org/F4698D30-CE68-4ACB-8931-577DAF817827

Figs 13–16 , 23–24 , 25–26 , 34 , 42 , 50 , 51 , 52 Japanese name: Kimon-tsumaorega

#### Type material.

***Holotype*: Japan**: 1♂, Tokyo Met., Ogasawara Isls., Chichijima Is., Asahiyama, Host coll. on 9. III. 2023, Emrg. on 4. IV. 2023, Host: dead wood, J.-H. Park leg., genitalia slide no. JP-325, ELKU. ***Paratypes*; Japan: [Tokyo**]: 1♀, Izu Isls., Hachijojima Is., Mihara-rindo, alt. 300 m, 19. X. 2023, LT, K. Hirai leg., DNA sample JHP-209, ELKU • [**Ogasawara Isls.]: [Nakodojima Is.**]: 4♂2♀, 16. VII. 2024, SW, J.-H. Park leg., JHP-208(♂), ELKU • 2♂4♀, same locality, 16. VII. 2024, S. Yagi leg., ELKU • [**Ototojima Is.**]: 2♂3♀, 28. IX. 2023, daytime research, Y. Matsui leg., JHP-205 (♀), ELKU • 1♂10♀, 12–13. VII. 2024, J.-H. Park leg., JP-324(♀) ELKU • 1♂, same locality, 12. VII. 2024, Y. Matsui leg., ELKU • [**Anijima Is.**]: 1♂1♀, alt. 162 m, 20. VI. 2022, Beating: dead leaves of Livistona chinensis var. boninensis, S. Tomura leg., JP-170(♀), JHP-277(♀), museum ID ELKU-I-L-Bonin 000077(♀), ELKU • 1♂, same locality, 20. VI. 2022, S. Yagi leg., JP-169, JHP-276, ELKU-I-L-Boonin 000076, ELKU • 1♂, North of C Line, Host coll. on 11. III. 2023, Emrg. on 6. IV. 2023, Host: Gahnia aspera, S. Yagi leg., JHP-105, ELKU • 2♀, Mt. Omaru-yama, 10. VII. 2024, Sweeping, Yu Hisasue leg., ELKU • [**Chichijima Is.**]: 1♂, Asahiyama, 26. VI. 2022, Beating: dead leaves of Livistona chinensis var. boninensis or *Pandanus boninensis* S. Tomura leg., ELKU • 1♂, same data, JP-172, JHP-279, ELKU-I-L-Bonin 000079, ELKU • 1♀, same locality, Coll. on 26. VI. 2022, Emrg. on 1. V. 2023, Host: dead wood bark, JP-131, JHP-083, ELKU • 1♂, same locality, Host coll. on 9. III. 2023, Emrg. on 4. IV. 2023, Host: dead wood, J.-H. Park leg., ELKU • 1♂3♀, same locality, 11. VI. 2023, SW, J.-H. Park leg., ELKU • 1♀, Mikazukiyama, 13. VI. 2023, M. Kimura leg., ELKU • 2♂4♀, same locality, 13. VI. 2023, J.-H. Park leg., JHP-086(♂), museum ID ELKU-I-L-000186(♀), ELKU • 1♂, Ohgiura, 18–31. VII. 2023, N. Tsuji leg., ELKU • 1♂, Hatsuneura, Larvae coll on 19. VI. 2022, Emrg. on 16. IX. 2022, Host: bark + fungi, S. Tomura leg., ELKU• 1♀, Higashidaira, 26. VI. 2022, T. Hirowatari leg., JP-019, DNA sample JHP-275, Museum ID ELKU-I-L-Bonin 000074, ELKU • 1♀, same locality, Host coll. on 10. III. 2023, Emrg. on 27. III. 2023, Host: dead wood, J.-H. Park leg., ELKU • 1♀, Tatsumi road, 11. VI. 2023, LT, J.-H. Park leg., ELKU • 1♂, Mt. Yoake-yama, Host coll. on 9. III. 2023, Emrg. on 10. V. 2023, Host: rotten wood, J.-H. Park leg., JP-130, JHP-085, ELKU • 1♂, Ôgamiyama, 11. VII. 2024, daytime research, Y. Matsui leg., ELKU • 1♂, same locality, 12. VII. 2024, Y. Matsui leg., ELKU • 2♂7♀, same locality, 11–16. VII. 2024, MT, J.-H. Park leg., ELKU • [**Hahajima Is.**]: 1♂1♀, Minamizaki, 23. VI. 2022, Beating: dead leaves of Livistona chinensis var. boninensis or *Pandanus boninensis*, S. Tomura leg., JP-327, ELKU • 2♂4♀, same locality, 10–11. XI. 2022, SW, J.-H. Park leg., JHP-123(♀), ELKU • 1♂, same locality, Host coll. on 10. XI. 2022, Emrg. on 20. III. 2023, Host: brown fungi, J.-H. Park leg., ELKU • 2♀, same locality, 10–11. XI. 2022, T. Hirowatari leg., ELKU • 1♀, same locality, 12. XI. 2022, T. Hirowatari leg., ELKU • 1♀, same locality, 15. III. 2023, S. Yagi leg., ELKU-I-L-000185 ELKU • 1♀, Funamidai, Host coll. on 21. VI. 2022, Emrg. on 1. VIII. 2022, Host: bark of stump, S. Tomura leg., ELKU • 1♂, same data, Emrg. on 9. IX. 2022, ELKU • 1♀, Funakiyama, 24. VI. 2022, S. Tomura leg., Beating: dead leaves of Livistona chinensis var. boninensis or *Pandanus boninensis*, JP-171, JHP-278, ELKU-I-L-Bonin 000078, ELKU • 1♂, same locality, 25. IX. 2023, daytime search, Y. Matsui leg., ELKU • 1♀, Funakiyama chûfuku, 16. VI. 2023, LT, J.-H. Park leg., ELKU • 2♀, Mt. Chibusa-yama, 9. XI. 2022, SW, J.-H. Park leg., JHP-124(♀), ELKU • 1♀, Nagahama tunnel, Coll. on 9. XI. 2022, Emrg. on 24. I. 2023, J.-H. Park leg., ELKU • 1♂, Mt. Chohki-yama, 22. VI. 2022, LT, S. Tomura leg., ELKU • 1♀, same locality, 15. VI. 2023, LT, J.-H. Park leg., ELKU • 2♀, Shinyûhigaoka, 14. VI. 2023, LT, J.-H. Park leg., ELKU • 1♀, Higashikou, 9. XI. 2022, SW, J.-H. Park leg., JHP-125, ELKU • 5♂, Kitakou, 16. VI. 2023, T. Hirowatari leg., ELKU • [**Mukohjima Is.**]: 1♂1♀, 15. VI. 2023, T. Hirowatari leg., JP-182(♂), JHP-104(♂), ELKU • 3♂3♀, same data, SW, S. Yagi leg., ELKU • 7♀, same data, J.-H. Park leg., ELKU • 1♀, same data, JP-181, JHP-103, ELKU • 3♂1♀, same data, Y. Matsui leg., JHP-204 (♀), ELKU • 1♀, 16. VII. 2024, daytime research, Y. Matsui leg., ELKU • [**Hirajima Is.**]: 3♂2♀, 23. IX. 2023, Y. Matsui leg., daytime search, JHP-206(♂), ELKU • [**Minami-iwoto Is.**]: 1♂1♀, alt. 500 m, 17. VI. 1982, M. Sato leg., Yutaka Arita Collection NSMT Donation, 2004, JP-195(♀), 196(♂), NSMT.

**Figures 25, 26.**
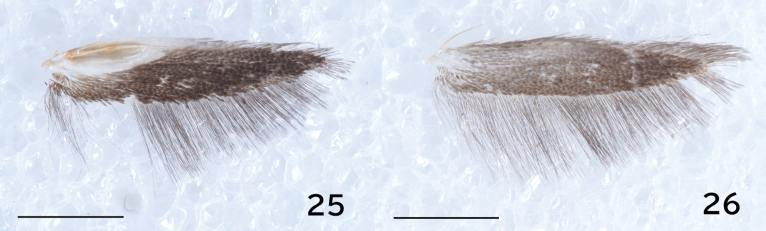
*Erechthias flavimacula* sp. nov., hindwing; 25. Male; 26. Female. Scale bars: 1.0 mm.

**Figures 27–30.**
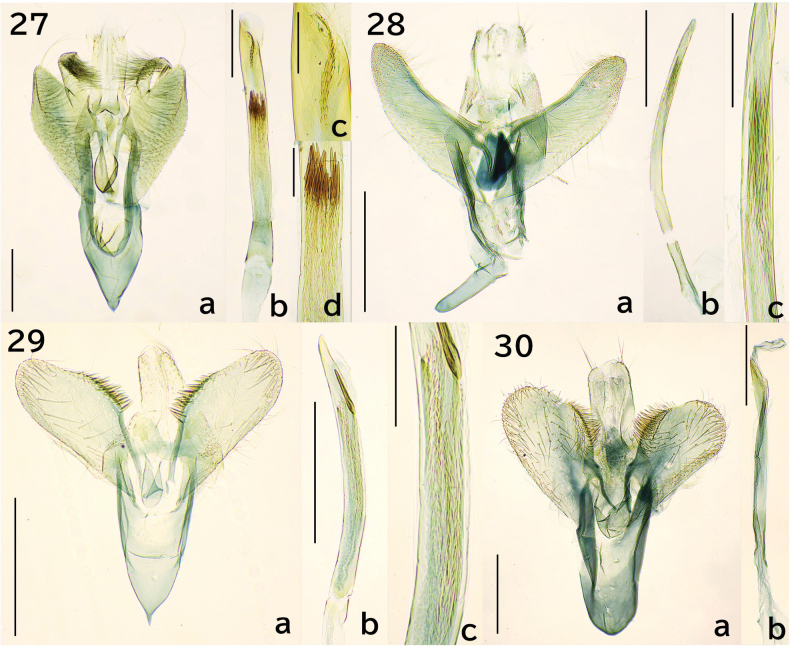
Male genitalia. 27. *Erechthias itoi* Moriuti & Kadohara, 1994, genitalia slide no. JP-164; 27a. Overview except phallus; 27b, 27c, 27d. Phallus; 28. *E. zebrina* (Butler, 1881), JP-328; 28a. Overview except phallus; 28b, 28c. Phallus; 29. *E. minuscula* (Walsingham, 1897), JP-309; 29a. Overview except phallus; 29b, 29c. Phallus; 30. *E. atririvis* (Meyrick, 1931), JP-305; 30a. Overview except phallus; 30b. Phallus. Scale bars: 0.3 mm (27a, 27b, 28a, 18b, 29a, 29b, 30a, 30b), 0.1 mm (27c, 27d, 28c, 29c).

**Figures 31–34.**
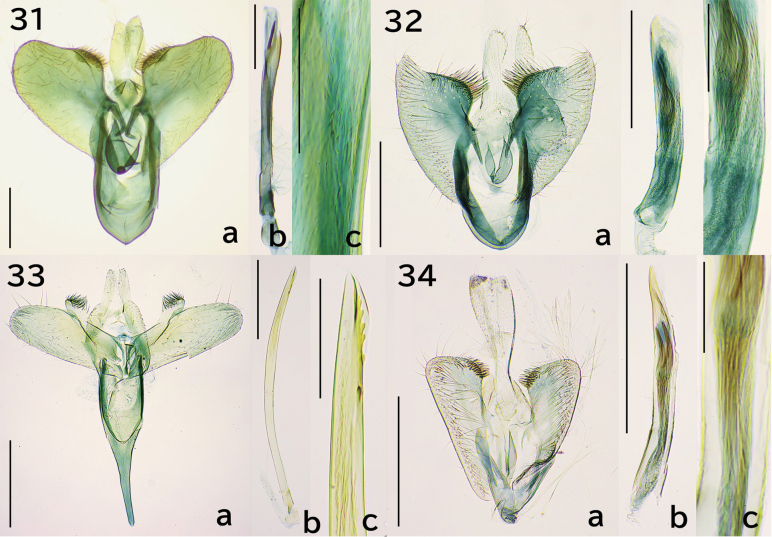
Male genitalia. 31. *Erechthias mirabilis* sp. nov., holotype, genitalia slide no. JP-312; 31a. Overview except phallus; 31b, 31c. Phallus; 32. *E. nidumicola* sp. nov., holotype, JP-310; 32a. Overview except phallus; 32b, 32c. Phallus; 33. *E. oculus* sp. nov., holotype, JP-128; 33a. Overview except phallus; 33b, 33c. Phallus; 24a. *E. flavimacula* sp. nov., holotype, JP-325; 24a. Overview except phallus; 24b, 24c. Phallus. Scale bars: 0.3 mm (31a, 31b, 32a, 32b, 33a, 33b, 34a, 34b), 0.1 mm (31c, 32c, 33c), 0.05 mm (34c).

**Figures 35–38.**
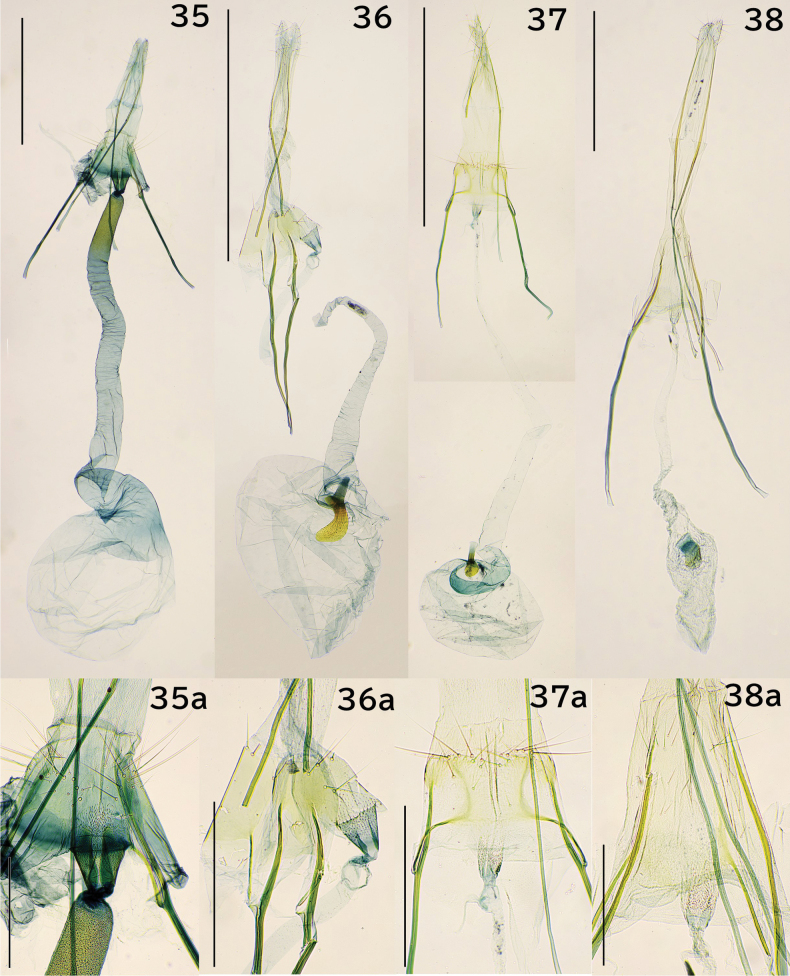
Female genitalia. 35. *Erechthias itoi* Moriuti & Kadohara, 1994, genitalia slide no. JP-138; 35a. Segment VIII; 36. *E. zebrina* (Butler, 1881), JP-304; 36a. Segment VIII; 37. *E. minuscula* (Walsingham, 1897), JP-315; 37a. Segment VIII; 38. *E. atririvis* (Meyrick, 1931), JP-306; 38a. Segment VIII. Scale bars: 1.0 mm (35–38), 0.3 mm (35a–38a).

**Figures 39–42.**
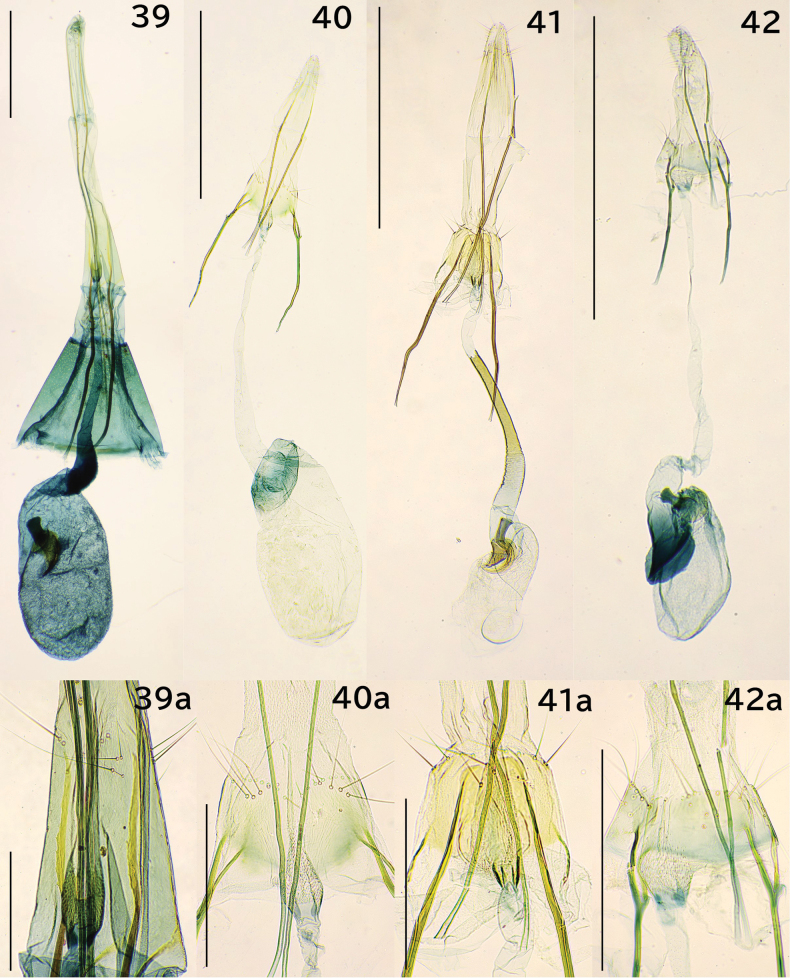
Female genitalia. 39. *Erechthias mirabilis* sp. nov., paratype, genitalia slide no. JP-018; 39a. Segment VIII; 40. *E. nidumicola* sp. nov., paratype, JP-141; 40a. Segment VIII; 41. *E. oculus* sp. nov., paratype, JP-317; 41a. Segment VIII; 42. *E. flavimacula* sp. nov., paratype, JP-131; 42a. Segment VIII. Scale bars: 1.0 mm (39–42), 0.3 mm (39a–42a).

#### Diagnosis.

The new species is externally similar to *E. epispora* (Lower, 1905), *E. nidumicola* sp. nov., and *E. oculus* sp. nov., but it can be distinguished by a combination of the following characteristics: the costal margin of the forewing with one or two narrow to broad triangular cream spots; the frenulum is a pair of slender bristles in females; the hindwing has secondary sexual scaling in males; the valva is relatively long, costal margin lacks lobe in the male genitalia; the signum is simple hook-shaped, and the corpus bursae is twisted and weakly sclerotized at the posterior 1/4 in the female genitalia.

#### Description.

**Male.** (Figs 13, 14, 23, 25) Forewing length 3.6 mm, antenna length 2.6 mm in holotype. Forewing length 3.2–4.1 mm (*n* = 5), antenna length 2.1–3.0 mm (*n* = 4) in paratypes. ***Head*.** Vertex cream to ocherous white, frons lighter than vertex; vertex with or without narrow gray bands. Labial palpus cream, second palpomere with robust brush hair, outer surface covered with dark brown to gray scales, and with few black bristles; basal 1/2 of third palpomere dark brown to gray. Antenna filiform; scape cream to ocherous white, notch absent; flagellomere cream, outer surface gray. ***Thorax*.** Mesonotum dark brown, posterior tip yellow. Tegula covered with brown to dark brown and yellow scales. Foreleg dark gray, inner surface cream; distal end of tarsus with cream band. Midleg cream; outer surface of tibia and tarsus ocherous gray. Hindleg cream, outer margin of tibia strongly concaved, and bearing with long ocherous gray hairs; tarsus ocherous gray, distal end cream. Forewing venation with Sc, R1–3 and R4+5, M1, M2+3, CuA1, CuA 2 and CuP, 1A+2A present; R1 from basal 3/7 of discal cell to 4/7 of costa; 1A fused with 2A from basal 1/4 of wing to end. Apex of forewing not upturned. Forewing ground color dark brown with cream to yellow oblique spots; costal margin with four trapezoidal spots: basal 1/3, middle, subapical area, and apex; dorsum with two spots: basal 1/8 and 2/3; submarginal area of termen and apex cream, fringed with black scales; cilia gray on outer margin, underside dark brown. Hindwing venation with Sc+R1, Rs, M1–3, CuA1–2 and CuP, 1A+2A present; Rs reduced and fused with Sc+R1, terminating on costa at apical 1/3; M1 and M2 stalked at basal 2/3 of wing; M1 terminating on costa at subapex; Sc and R modified to accommodate secondary sexual scaling; frenulum a single slender bristle. Hindwing fuscous brown to gray; cilia fuscous brown to gray; base of Sc and R with secondary sexual scaling. ***Abdomen*.** covered with dark brown to fuscous brown scales.

**Female.** (Figs 15, 16, 14, 26, 50) Forewing length 3.3–5.0 mm (*n* = 11), antenna length 2.1–3.1 mm (*n* = 8) in paratypes. Almost same as male but different as follows: tibia not concaved in hind leg; yellow spots tend to be wider than male in forewing; Sc+R1 not modified, Rs terminating on costa at apical 1/5 in hindwing vein; hindwing simple, not with secondary sexual scaling; frenulum consisting of two slender bristles.

**Male genitalia.** (Fig. 34) Uncus long, a pair of membranous lobes, apex with few bristles. Tegumen narrow ribbon-shaped, fused with vinculum. Vinculum almost same length of tegumen; saccus rounded triangle. Valva elongated narrow trapezoidal with rough bristles at ventral and anterior margins; ventral margin straight, apically rounded; dorsal costa broad, with short spines, basicostal process absent. Juxta strongly sclerotized, elongated and narrow pouched. Phallus thick cylindrical, weakly curved, 1.4 × length of valva; vesica with one pair of twisted, sharp, blade-shaped cornuti and a few small spicular cornuti.

**Figures 43–50.**
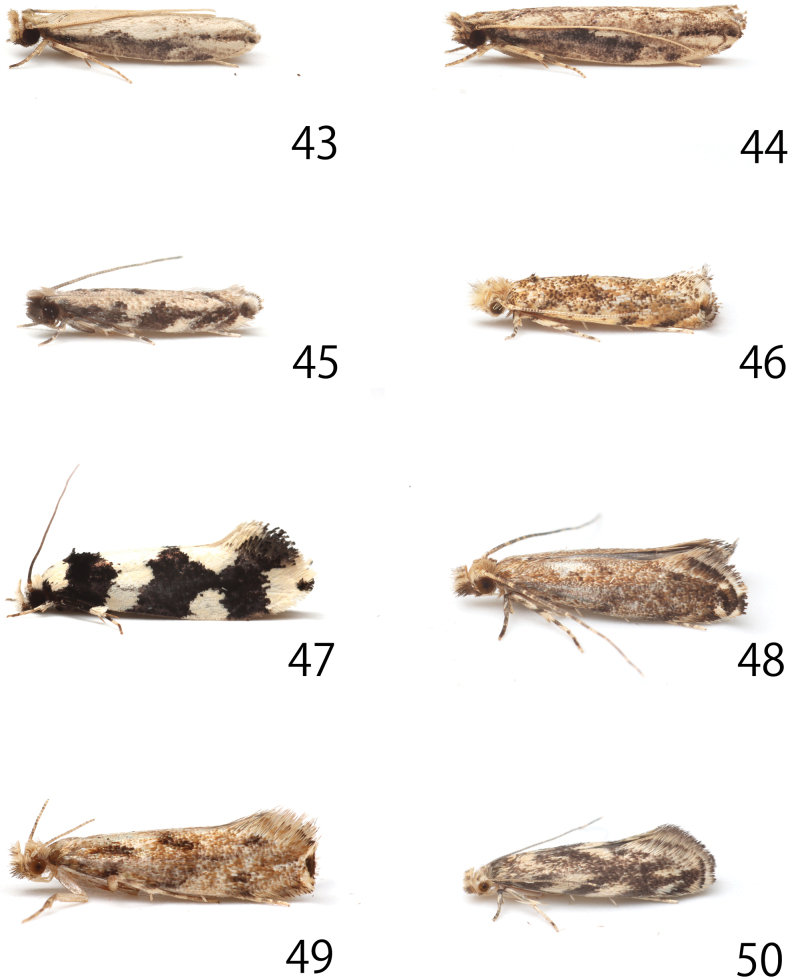
Resting position. 43. *Erechthias itoi* Moriuti & Kadohara, 1994, male; 44. Ditto, female; 45. *E. zebrina* (Butler, 1881); 46. *E. minuscula* (Walsingham, 1897); 47. *E. mirabilis* sp. nov., female; 48. *E. nidumicola* sp. nov. male; 49. Ditto, female; 50. *E. flavimacula* sp. nov.

**Figure 51.**
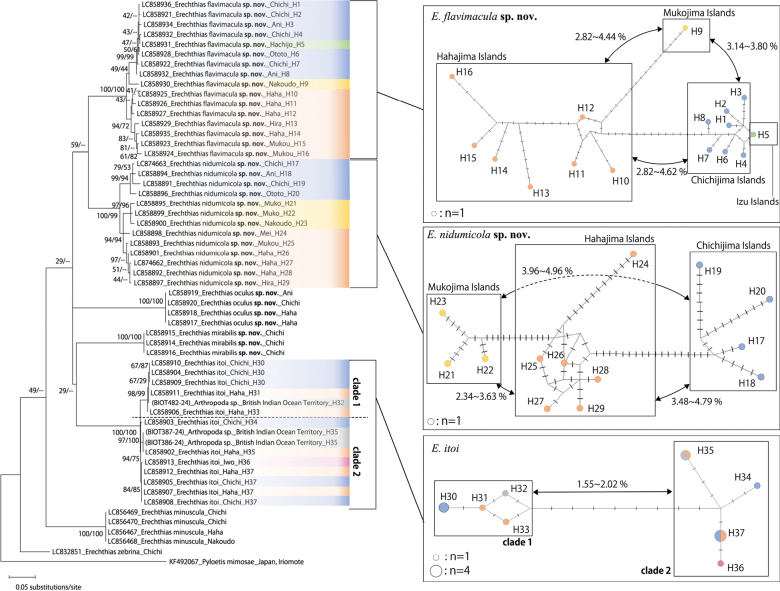
Maximum likelihood trees of *Erechthias* spp. from Ogasawara islands and haplotype networks of *E. nidumicola* sp. nov., *E. flavimacula* sp. nov. and *E. itoi*. Branch lengths of the phylogenetic tree are proportional to the genetic distances and the scale bar indicated 0.05 substitutions/site. Highlights in the phylogenetic tree and haplotype colors in the network correspond to sampling sites on the phylogenetic tree (Blue: Chichijima Islands; Red: Hahajima Islands; Yellow: Mukojima Islands; Pink: Iwoto Islands; Green: Izu islands; Gray: British Indian Ocean Territory). The number near the node is the bootstrap value (ML/MP). Nodes annotated by “—” indicate a mismatch in the topology between the ML and MP methods. The sizes of the circles are proportionate to the numbers of specimens sharing a particular haplotype. The arrow marks and “%” numbers are indicated to the genetic distances between each haplotype groups.

**Female genitalia.** (Fig. 42) Ovipositor broad, 3 × length of segment VIII; papillae analis with rough short bristles; apophysis posterioris slightly longer than apophysis anterioris. Segment VIII broad, rectangular, posterior margin with ~20 long bristles; tergum VIII with stout dorsal rami fusing with apophysis anterioris; ostium located at anterior margin of sternum VIII; ostium wide, funnel-shaped with many small spines, posterior end weakly sclerotized. Ductus bursae tubular, 2.3 × length of corpus bursae; at anterior 1/4 broad and curved. Corpus bursae piriform, posterior end weakly sclerotized; signum spatulate.

#### Distribution.

Japan (Hachijohjima Is., Nakodojima Is., Ototojima Is., Anijima Is., Chichijima Is., Hahajima Is., Mukohjima Is., Hirajima Is., Minami-iwoto Is.).

#### Biology.

Few adults emerged from *Ganoderma orbiforme* (Fr.) Ryvarden (Polyporales: Ganodermataceae), white fungi (Polypoaceae), litter, wood bark, and an ear of *Gahnia aspera* (R.Br.) Spreng (Poales: Cyperaceae). Adults were collected in June, July, September, and November from the Ogasawara Islands and in October from the Izu Islands.

**Figure 52.**
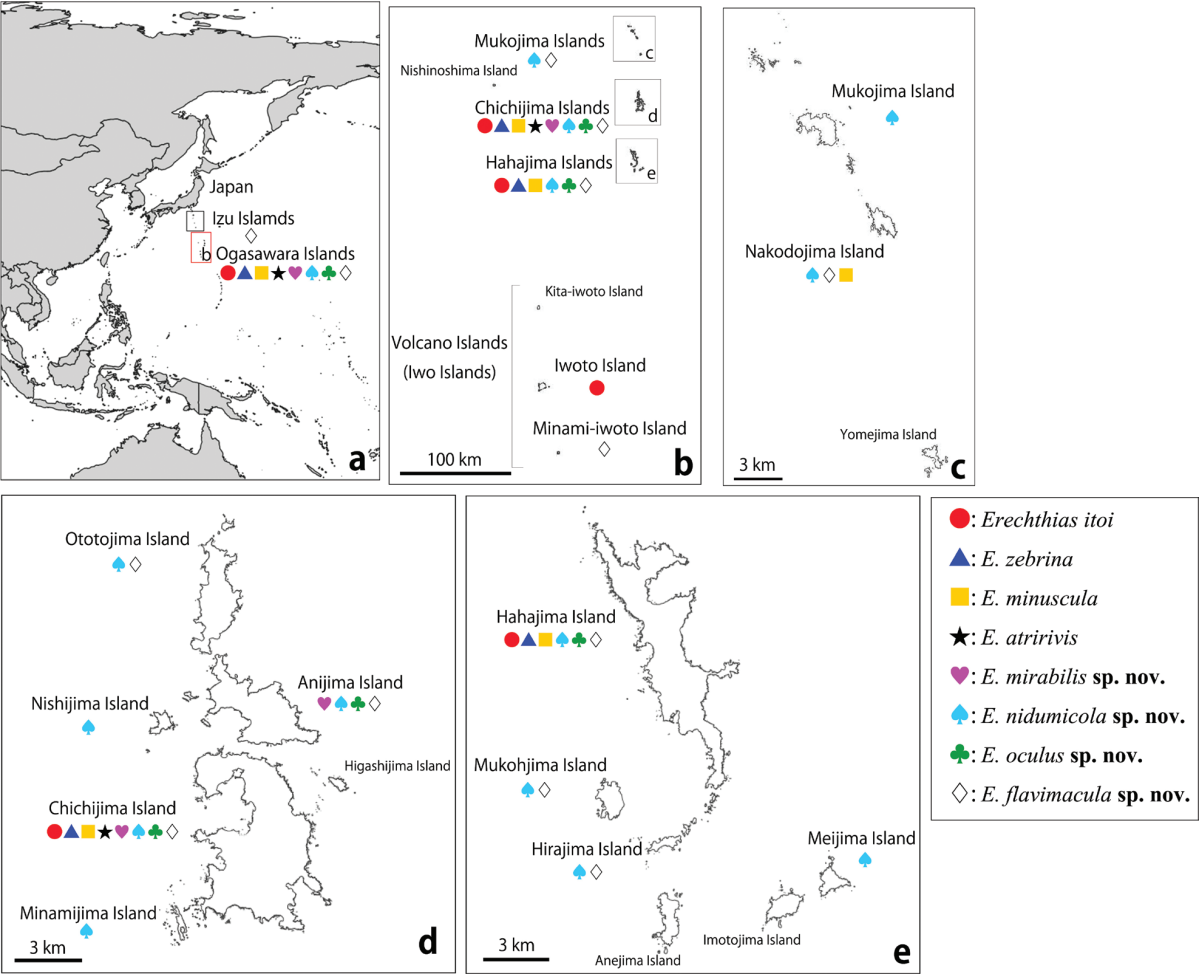
Distribution map of *Erechthias* spp. a. Location of the Ogasawara Islands; b. Detail of the Ogasawara Islands; c. Detail of the Mukojima Islands; d. Detail of the Chichijima Islands; e. Detail of the Hahajima Islands.

#### Etymology.

The name of the new species is derived from the Latin *flavus* (yellow) + *macula* (spots), referring to the forewing pattern consisting of many yellow spots.

#### DNA analyses.

The intraspecific pairwise distances of this species were 0.03%–4.41% (*n* = 16) (Suppl. material 2). The intraspecific genetic structure was analyzed using 16 samples, and a haplotype network was constructed (Fig. 51). All the samples had unique haplotypes.

#### Remarks.

This species is endemic to the Izu-Ogasawara Islands. In a few female specimens, the yellow spots become extremely wide, and the forewings almost become yellow (Fig. 15). Many specimens were obtained by beating dead leaves of Livistona chinensis var. boninensis and *Pandanus boninensis* during the day.

## ﻿Discussion

Two endemic new species, *E. nidumicola* sp. nov., and *E. flavimacula* sp. nov., showed high genetic divergence, with some samples with genetic distances exceeding the COI threshold (2–3%), proposed by Hebert et al. (2003) for the recognition of cryptic species. Haplotypes were divided into three units corresponding to each group of islands, except for one haplotype from Izu Islands (Fig. 51). These genetic distances suggest that the populations on each island group might represent independent species. However, we could not detect any morphological differences among them. Therefore, in this study, these genetically diverse populations were treated as a single species, but depending on the results of future studies, this taxonomic treatment may need to be reconsidered.

In these two species, the arrangement of each haplotype group differed from the order of the islands, with the Hahajima Islands group in the middle, from which the Chichijima Islands group and the Mukojima Islands group branched off separately. From a simplistic point of view, it seems that these species were first established on the Hahajima Islands group and then spread to other islands in the archipelago. However, we only had 29 samples of a partial COI region, all of which showed unique haplotypes, with many haplotypes missing. Thus, further research with additional sampling, such as microsatellite-based analysis, estimation of fixation indices, and more detailed analyses, is needed to clarify the genetic structure and dispersal route.

In *E. itoi*, haplotypes were roughly divided into two groups; however, they were not grouped by archipelago and did not reflect geographical relationships (Fig. 51). Currently, this species is only known from populated islands in the Ogasawara Islands. We conducted daytime and nighttime field surveys and set traps on uninhabited islands but were unable to confirm their presence. In addition, the Hachijojima Island samples of *E. flavimacula* sp. nov. formed a group with the Chichijima Islands group rather than with the Mukojima Islands group, which is geographically closer (Fig. 51). On the way to the Ogasawara Islands aboard the cargo and passenger ship “Ogasawara-maru,” we collected some microlepidoptera while sailing through the Izu Islands Sea area that had not been recorded in the Ogasawara Islands (Park et al. 2025). The distribution pattern of the haplotypes of these species suggests the possibility of genetic exchange between the islands due to human migration.

Some island-endemic *Erechthias* have unique biology, such as leaf miners, lichenophagous, and portable case-making behavior (Marler and Muniappan 2006; Davis and Mendel 2013). Among the species discovered in this study, *E. nidumicola* sp. nov. used bird nests and fungi, whereas *E. flavimacula* sp. nov. used various habitats and rotten organic matter. Adaptation to new niches may have contributed to the adaptive radiation of endemic species in this genus.

## Supplementary Material

XML Treatment for Erechthias

XML Treatment for Erechthias itoi

XML Treatment for Erechthias zebrina

XML Treatment for Erechthias minuscula

XML Treatment for Erechthias atririvis

XML Treatment for Erechthias mirabilis

XML Treatment for Erechthias nidumicola

XML Treatment for Erechthias oculus

XML Treatment for Erechthias flavimacula

## ﻿Additional information

### Use of AI

No use of AI was reported.

### Author ORCIDs

Jinhyeong Park https://orcid.org/0009-0005-3121-9990

Sadahisa Yagi https://orcid.org/0000-0002-4261-1219

Toshiya Hirowatari https://orcid.org/0000-0002-6839-2229

### Data availability

All of the data that support the findings of this study are available in the main text or Supplementary Information.

## Supplementary materials

Supplementary material 1 Names and collection localities for taxa used in phylogenetic analyses and calculating the genetic distance and haplotype network Data type xlsx https://binary.pensoft.net/file/1402236This dataset is made available under the Open Database License (http://opendatacommons.org/licenses/odbl/1.0/). The Open Database License (ODbL) is a license agreement intended to allow users to freely share, modify, and use this Dataset while maintaining this same freedom for others, provided that the original source and author(s) are credited.Jinhyeong Park, Sadahisa Yagi, Toshiya Hirowatari

Supplementary material 2 Genetic distances of the partial COI gene sequences of *Erechthias* spp. (Ogasawara Islands, Hachijo Island (Izu Islands)) and Arthropoda sp. from Indian Ocean Data type xls https://binary.pensoft.net/file/1402237This dataset is made available under the Open Database License (http://opendatacommons.org/licenses/odbl/1.0/). The Open Database License (ODbL) is a license agreement intended to allow users to freely share, modify, and use this Dataset while maintaining this same freedom for others, provided that the original source and author(s) are credited.Jinhyeong Park, Sadahisa Yagi, Toshiya Hirowatari
